# From Light to Virtual: Comparing RTI and VRTI for Ichnological Analysis

**DOI:** 10.3390/jimaging12090428

**Published:** 2026-09-09

**Authors:** Francesca Fabbri, Alice Bordignon, Luisa Ammirati, Michela Contessi

**Affiliations:** 1 Department of Classical Philology and Italian Studies, University of Bologna, 40126 Bologna, Italy; alice.bordignon2@unibo.it (A.B.); luisa.ammirati2@unibo.it (L.A.); 2 Geology Collection “Giovanni Capellini Museum”, University Museum System, University of Bologna, 40126 Bologna, Italy; michela.contessi@unibo.it

**Keywords:** Reflectance Transformation Imaging (RTI), Virtual Reflectance Transformation Imaging (VRTI), ichnology, digital cultural heritage, 3D documentation, FAIR workflows, photogrammetry, structured-light scanning, usability evaluation, perceptual transparency

## Abstract

Reflectance Transformation Imaging (RTI) has long been employed across multiple research domains to document surface textures, inscriptions, and fine morphological features, including paleontology. However, RTI requires specialized equipment and controlled acquisition protocols that are often impractical in fieldwork or heterogeneous documentation contexts. To overcome these limitations, Virtual Reflectance Transformation Imaging (VRTI), based on high-resolution 3D models acquired through photogrammetry or structured-light scanning, has emerged as a promising alternative. This study investigates whether VRTI offers an operationally simpler yet perceptually consistent alternative to conventional RTI for dinosaur footprint interpretation, while providing a standardized FAIR-compliant VRTI workspace implemented in an open-source 3D environment. A comparative protocol was developed and validated through a classroom experiment involving paleobiology students, who performed footprint delineation and morphological interpretation using both RTI and VRTI datasets derived from a dinosaur footprint from the Geological Collection of the “Giovanni Capellini Museum” of the University of Bologna. The evaluation combines quantitative statistical analysis with qualitative expert assessment to compare interpretative performance, while optimal PTM-based raking-light conditions are also investigated. The results of the statistical comparison between RTI and VRTI remained non-significant (*p* > 0.05) for the tasks involved in the experiment, indicating comparable interpretative performance between the two approaches. Building on this validation, a preliminary usability assessment of the proposed workspace (*n* = 11) yielded a mean System Usability Scale (SUS) score of 67.7, supporting its practical applicability while providing a reproducible, interoperable, and scalable workflow for cultural heritage documentation.

## 1. Introduction

Reflectance Transformation Imaging (RTI) has long been employed across several research fields. In paleontology, it has proven particularly effective for the study of small specimens and dinosaur footprints, allowing researchers to identify morphological features that are not visible to the naked eye [1]. The interpretation of dinosaur footprints for documentation purposes remains challenging because subtle morphological features are often difficult to distinguish consistently, both in the field and in digital 3D models. This is caused by the inherent difficulty in reliably discerning track morphology both on-site and within digital 3D models. The use of raking light in combination with 2D imaging techniques enhances morphological detail and reveals microstructures that would otherwise remain undetected, which is effective for the study of small fossils. Among these imaging techniques based on raking illumination, Reflectance Transformation Imaging (RTI) has become one of the most widely adopted approaches. However, RTI relies on specialized equipment, such as a light dome or at least a camera mounted on a tripod, as well as a controlled acquisition environment. The method requires complete darkness during image capture and the object to remain entirely stationary throughout the acquisition process to ensure the quality of the resulting dataset. These constraints often limit its practical application. In excavation fields, RTI acquisition is frequently hindered by uncontrolled lighting conditions, including diffuse illumination and changing shadows, which prevent reliable image capture. Field campaigns are often conducted in remote locations where transporting and powering specialized RTI equipment may be unrealistic; moreover, conventional RTI is inherently difficult to apply to large or continuous track-bearing surfaces, for which dome-based acquisitions are impractical. In museum environments, the need to minimize ambient lighting and maintain a completely static acquisition setup is often incompatible with exhibition galleries, while acquisitions through display cases are further complicated by reflections that vary with the changing illumination angle. These limitations have encouraged the development of Virtual RTI (VRTI), in which the acquisition process is conducted within a virtual environment using 3D models. VRTI reproduces the principles of RTI within a digital 3D environment, allowing interactive relighting directly on reconstructed surfaces without requiring a physical acquisition setup. Furthermore, when no RTI dataset was collected during the original digitization campaign, conventional RTI cannot be generated retrospectively, whereas VRTI enables interactive relighting directly from pre-existing digital assets. Despite its growing adoption, previous VRTI studies have employed heterogeneous and case-specific implementations [2,3,4,5], and a standardized and validated workflow, including an assessment framework for direct comparison between RTI and VRTI, is still lacking. As detailed in the Section 2, methodological variability mainly concerns the 3D digitization techniques employed, the configuration of the virtual lighting environment, and rendering parameters. This operational heterogeneity directly impacts both workflow setup and rendering consistency, ultimately limiting reproducibility, transparency, and comparability across studies. 

To address this challenge, a dedicated VRTI workspace was developed within the open-source software Blender (https://www.blender.org/, accessed on 1 July 2026) to implement a standardized workflow for virtual relighting and image generation. This development enables the investigation of the primary scientific objective of this work, which is to determine whether VRTI can provide an operationally simpler alternative to conventional RTI for dinosaur footprint interpretation with no detectable differences in perceptual performance. To this end, the proposed workflow is first validated through a controlled comparative protocol designed to evaluate footprint readability and perceptual transparency between RTI and VRTI. The methodology is assessed through a case study involving a dinosaur footprint from the Geological Collection of the “Giovanni Capellini Museum” of the University Museum Network, University of Bologna. Following the validation of the proposed methodology, the usability of the Blender workspace is evaluated through a pilot System Usability Scale (SUS) study to assess its suitability as a standardized FAIR-compliant digital asset for VRTI analysis.

The objectives of this study are, therefore, as follows:

- To develop and implement a VRTI workflow through a dedicated virtual workspace in Blender, providing a reproducible environment for virtual relighting and image generation;
- To validate the scientific reliability of the proposed VRTI methodology by assessing whether it provides an operationally simpler alternative to conventional RTI for dinosaur footprint interpretation while yielding comparable perceptual performance through a controlled comparative protocol;
- To evaluate the usability of the validated workspace through a pilot System Usability Scale (SUS) study, assessing its suitability for adoption by cultural heritage and ichnological practitioners.

## 2. Related Works

### 2.1. Introduction to the Study of Footprints and Documentation Problems

Ichnology, the study of fossil tracks and traces, is a discipline born in the 1830s, when Edward Hitchcock first described some dinosaur tracks in the Jurassic sediments of Massachusetts, USA [6]. Tracks are three-dimensional surfaces that can be found as positive relief or negative depressions. Since the beginning, it has been difficult to represent tracks in an objective way, and Hitchcock himself used different types of drawing, such as outlines or blended black and white, to underline the diagnostic characteristics of tracks [7]. Regarding the difficulties in digging most of the track sites, in the XIX century, many efforts were made to extract track slabs from the fields and exhibit them in many museums, where they are still available to researchers. However, since the XX century, this practice has been mostly abandoned due to the elevated costs and increasing difficulties in reaching the newly discovered sites. Since then, molding, photography and hand sketches have been the only way to document most ichnosites. Most of the bibliography up to the 1990s still relies on black-and-white photos and track outlines, which represent interpretative reconstructions of the original data and are, therefore, strongly influenced by the researcher’s skill in depicting them [8].

Since the new millennium, the digital revolution has greatly modified the study of ichnology; 3D scanning and photogrammetry techniques have become available to a wide range of researchers and institutions [9,10]. Nowadays, the creation of mathematically based 3D models of tracks provides an easier and more objective way to correlate data if the models and the associated acquisition metadata are available. To address the need for standardized documentation, in [8], a protocol is proposed for tracks and traces of digitization and representation in scientific publications. Although this proposal is valid, tracks remain difficult to interpret both in the field and in digital models, and many details are difficult, if not impossible, to recognize under non-ideal light conditions. While track topography may preserve a direct record of the trackmaker’s anatomy, the final morphology of a track is more often influenced by complex foot–sediment interactions involving numerous variables. These include locomotion speed and substrate water content [11], making it challenging to identify the boundaries of the true track [12,13].

### 2.2. RTI and Virtual RTI: Limitations, Extensions, and Methodological Challenges

RTI is a mature imaging technique defined by the non-profit organization Cultural Heritage Imaging (https://culturalheritageimaging.org/, accessed on 1 July 2026) as “a computational photographic method that captures a subject’s surface shape and color and enables the interactive re-lighting of the subject from any direction, revealing details that are not visible to the naked eye” (https://culturalheritageimaging.org/Technologies/RTI/, accessed on 1 July 2026). 

RTI, along with other 2D imaging techniques, has long been used for documentation purposes across various research fields like archeology, numismatics, and paleontology. This technique enhances surface visibility by using controlled lighting to capture how light interacts with an object’s surface. It is typically carried out in two ways: by manually moving a light source around a stationary camera (RTI) and subject, or by using a dome fitted with fixed light sources that illuminate the object in sequence while a static camera records the images (HRTI—Hemispherical Reflectance Transformation Imaging) [14]. In both cases, it is preferable to have a dark lighting condition. While HRTI systematically samples illumination directions over a hemisphere, RTI provides more limited control over the origin of the light sources and the planning of the lighting configuration. However, HRTI is not always feasible due to the limited availability of appropriate equipment, as it requires a specialized dome that is often prohibitively expensive for museums and public institutions. In recent years, RTI has increasingly been combined with 3D models generated through techniques such as structure-from-motion (SfM) photogrammetry or structured-light scanning (SLS). This combination is primarily employed in the field of cultural heritage to document complex surfaces and enhance data readability. In ref. [15], a comparative analysis of the main documentation techniques used for lithic materials is provided, including 2D drawings, 3D models, and RTI, evaluating them according to factors such as cost, acquisition time, ease of implementation, and output quality. The authors argue that, where full 3D metrics are not essential, RTI is the most effective method to document individual lithic artifacts, offering the best compromise between cost, time, portability, objectivity, and detailed visualization of micro-relief. However, RTI presents several limitations and critical issues related to its scalability. The integration of RTI principles with 3D technologies has led to the development of Virtual Reflectance Transformation Imaging (VRTI), in which relighting is performed within a virtual environment using a three-dimensional representation of the documented surface. In the current state of the art, VRTI has primarily been explored as a means of extending RTI principles to contexts in which conventional acquisition is difficult or impractical, particularly large-scale or geometrically complex surfaces. At the same time, its methodological development remains heterogeneous, especially with regard to the validation of VRTI and the software environments and processing workflows adopted for its implementation. The following sections, therefore, examine these three interconnected aspects of VRTI research: its application to large-scale and complex contexts, the validation of VRTI methodologies, and the variability in software implementations and workflows. 

### 2.3. VRTI for Large-Scale and Complex Contexts

The primary limitation that has contributed to the development of VRTI is the difficulty of applying RTI to large-scale objects or extensive sites. Large-scale archeological sites or museum environments have lighting conditions and spatial constraints that are not easily adaptable, whereas RTI acquisition requires controlled lighting. To overcome these limitations, VRTI is increasingly used. In ref. [2], the structure-from-motion technique is used combined with VRTI for the documentation of carved inscriptions at ancient Telmessos (Fethiye), demonstrating its applicability in a complex urban archeological context characterized by large and inaccessible inscriptions and monument geometries that could cause self-occlusion during conventional RTI acquisition. This is supported by ref. [16], who successfully applied VRTI in underwater photogrammetry to survey 3D models of two granite blocks, demonstrating its suitability for challenging and constrained contexts. Virtual methodology is often preferred due to the opportunity to reuse existing 3D data [17]; combining an RTI Builder and the open-source 3D modeling software Blender enabled effective VRTI analysis of 3D data for graffiti documentation, here applied due to the large size of the Catacombs of San Gennaro. VRTI has also been proposed as a solution for documenting curved cultural heritage surfaces, where conventional RTI cannot be effectively applied because of geometric and logistical constraints. By combining terrestrial laser scanning, surface unrolling, and virtual relighting, the method extends RTI-based surface analysis to large non-planar objects [5]. These studies suggest that VRTI is particularly advantageous in contexts where conventional RTI is impractical, such as large-scale archeological sites, complex geometries, or environments where controlled illumination cannot be achieved. By decoupling the relighting process from physical acquisition, VRTI also enables the reuse of pre-existing 3D digital assets. 

### 2.4. Validation of VRTI Methodologies

While RTI/HRTI results mainly depend on camera resolution, illumination sampling, and dataset density, VRTI outcomes also depend on the resolution and quality of the underlying 3D model. The 3D acquisition technique, therefore, represents an additional methodological variable, as differences in spatial resolution, geometric accuracy, and surface coverage can affect the representation of morphological features subsequently examined through virtual relighting. However, these acquisition-related variables are not specific to VRTI but correspond to those affecting 3D digitization more generally and are primarily determined by the available equipment, the characteristics of the object or surface, and its scale. Accordingly, VRTI applications have relied on different acquisition approaches, primarily structure-from-motion (SfM) photogrammetry, structured-light scanning (SLS), and terrestrial laser scanning (TLS), selected according to the scale, geometry, accessibility, and documentation requirements of the target surface.

These techniques involve different practical and technical trade-offs. SfM photogrammetry is generally more portable and adaptable to large or difficult-to-access contexts and can, therefore, facilitate the acquisition of extensive surfaces, although the resulting geometry is dependent on image quality, surface texture, camera configuration, and reconstruction parameters. SLS can provide dense geometric data suitable for the documentation of fine surface morphology, but its application is generally more constrained by object size, accessibility, and reflective surfaces. Meanwhile, TLS is particularly suited to large-scale surfaces and architectural contexts. Since VRTI derives its relighting information from the geometry of the reconstructed surface, these acquisition-related differences may influence the visibility of subtle features and, therefore, need to be considered when evaluating VRTI performance [18]. In this respect, ref. [15] compared the 3D survey methodology and RTI in terms of acquisition time, showing that photogrammetric acquisition is significantly faster than RTI, suggesting that RTI is preferable only when no other morphological data are necessary. 

To overcome these physical and logistical constraints, VRTI translates this framework into a digital space; the core principle of VRTI is precisely the reconstruction of the RTI acquisition workflow and its physical environment within a virtual domain. 

As previously noted, VRTI can be applied to large and complex contexts where RTI is unfeasible. However, existing applications lack direct comparison with traditional RTI and focus mainly on rendering settings rather than methodological validation. For instance, ref. [4] applied VRTI and vPTM selectively to highly eroded petroglyphs when conventional inspection of the 3D models provided insufficient morphological readability. While demonstrating the effectiveness of virtual relighting for enhancing poorly preserved petroglyphs, their comparative assessment primarily concerned different 3D acquisition and visualization techniques, rather than comparing physical RTI and VRTI. Similarly, ref. [2] explicitly compared VRTI with conventional H-RTI, assessing its effectiveness and practical advantages as a field-recording method. However, their comparison remained primarily qualitative and application-oriented, without evaluating whether the two techniques lead users to equivalent morphological interpretations.

A similar approach was adopted in ref. [3], which evaluated the feasibility of VRTI by generating both RTI and VRTI datasets from the same small test object. Their workflow followed a controlled workflow:

same object → RTI acquisition + SfM acquisition → V-RTI generation → same ROI → same RTI enhancement → same lighting/export parameters → side-by-side visual comparison.

By comparing corresponding regions of interest (ROIs) under identical visualization modes (Diffuse Gain, Coefficient Unsharp Mask, and Normal Map) and export parameters, the authors demonstrated that the VRTI workflow was technically capable of reproducing RTI visualizations under controlled conditions. 

However, the evaluation remained strictly qualitative and focused on the consistency of the generated outputs rather than investigating whether the two modalities provide perceptual equivalence or support comparable user interpretations.

A similar approach was adopted in ref. [5], where the methodology proposed for curved cultural heritage surfaces was evaluated through a qualitative assessment of the generated VRTI, demonstrating its capability to reveal surface features while discussing computational performance and workflow efficiency, rather than validating the equivalence of interpretation with conventional RTI.

Overall, the existing literature has established the technical feasibility of VRTI and demonstrated its utility in contexts where conventional RTI cannot be readily employed. Nevertheless, validation to date has been primarily based on qualitative visual assessment of the generated outputs. While this approach is appropriate for evaluating workflow performance, it does not determine whether VRTI provides the same perceptual support as conventional RTI for the identification and interpretation of morphological features. 

### 2.5. Software Implementations and Workflow Variability

**Figure 1:**
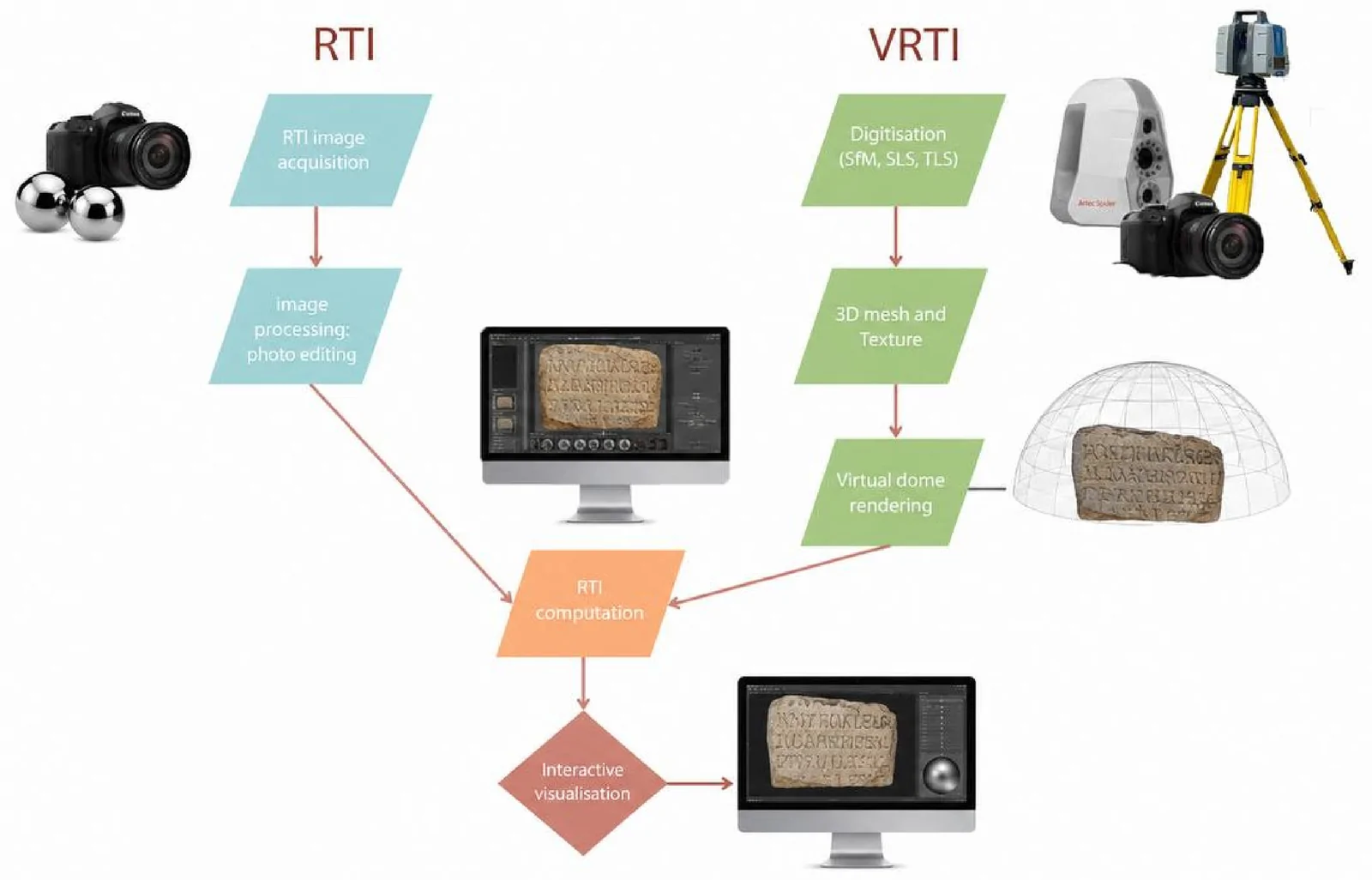
Schematic comparison of the main processing stages of conventional RTI and VRTI workflows.

The conventional RTI pipeline can be broadly divided into four main stages:

- Image acquisition: The object is photographed from a fixed camera position, typically mounted on a tripod, while the illumination direction is varied either manually or through a lighting dome. Reflective spheres are included in the scene to determine the light source positions.
- Image post-processing: RAW images are calibrated (e.g., for white balance and exposure) and exported in a suitable image format.
- RTI computation: Using RTI-building software to generate the reflectance file (e.g., .ptm or .rti).
- Interactive visualization: Finally, the final file is explored using an RTI viewer enabling interactive relighting and additional visualization modes (Figure 1) [19].

Based on the primary VRTI literature summarized in Table 1, VRTI implementations share a conceptual pipeline that encompasses five key stages:

3D digitization → 3D mesh and texture → virtual rendering → RTI computation → interactive visualization

- Three-dimensional digitization: Using photogrammetry, SLS, and TLS;
- Three-dimensional mesh and texture: Processing of the acquired 3D data to generate point clouds, mesh and texture computation, exported in a suitable 3D format;
- Virtual dome rendering: Import of the 3D model into the virtual environment and rendering under a virtual dome using multiple raking-light directions.
- RTI computation: Processing of the rendered image set using RTI-building software to generate the final reflectance file;
- Interactive visualization: Exploration and interactive relighting of the resulting file using an RTI viewer.

The main difference between RTI and VRTI lies in the first steps of the pipeline.

While the final stages of the workflow, namely, RTI building and RTI visualization, remain largely consistent across both modalities, greater variability occurs in the first two stages of VRTI. Existing studies employ diverse 3D capture technologies, distinct virtual software environments, and varying rendering configurations, as shown in Table 1 (e.g., dome geometry, light count and distribution, rendering engines, output resolutions, and levels of automation) [2,3,4,5,16,17]. 

Consequently, although the underlying principle of VRTI remains consistent across existing applications, there is currently no standardization in terms of workspace configuration, processing pipeline, software environment, parameter selection, or the information reported to ensure reproducibility. The reviewed studies generally report VRTI outputs as suitable for enhancing surface features and supporting their visual interpretation across different application contexts. However, output quality and reliability are assessed through different approaches, ranging from qualitative visual inspection and application-specific evaluation to direct comparison with conventional RTI, and no shared assessment approach clearly emerges across the reviewed studies. This methodological variability does not prevent the implementation of VRTI but makes direct comparison and reproduction across studies more difficult, supporting the need for a clearly documented and standardized workflow.

## 3. Materials and Methods

### 3.1. The Context

The specimens here studied using RTI are three dinosaur tracks donated by E. Hitchcock to Giovanni Capellini in 1863, at the end of his journey in North America [20]. The tracks were part of a collection of specimens from Massachusetts and Connecticut, now hosted at the Beneski Museum at Amherst College (Amherst, MA, USA), which included the first footprints ever described by Hitchcock [7]. Capellini visited Hitchcock and his collection in 1863, and received some fossil track samples from his assistant, Prof. Shepard. These specimens originated from the Connecticut Valley, specifically from the Turners Falls Formation, a unit of the North American Newark Supergroup, which comprises erosional remnants of rift-basin strata formed during the Lower Jurassic at the initial stages of the breakup of Pangea [21]. The tracks have been historically attributed to the ichnogenus Anomoepus, originally diagnosed as “bipedal in gait, the manus impressing only when seated: pes tetradactyl, but functionally tridactyl, digitigrade with an elongated metatarsal segment in evidence when the animal rests” [22,23]. More recently, Anomoepus has been described as an Early Jurassic footprint genus produced by a relatively small, gracile ornithischian dinosaur [24]. It has a pentadactyl manus and a tetradactyl pes, but only three pedal digits normally impressed while the animal was walking. Anomoepus is diagnosed by having the metatarsal phalangeal pad of digit IV of the pes lying in line with the axis of digit III, in combination with a pentadactyl manus [24]. 

### 3.2. RTI Acquisition

**Figure 2:**
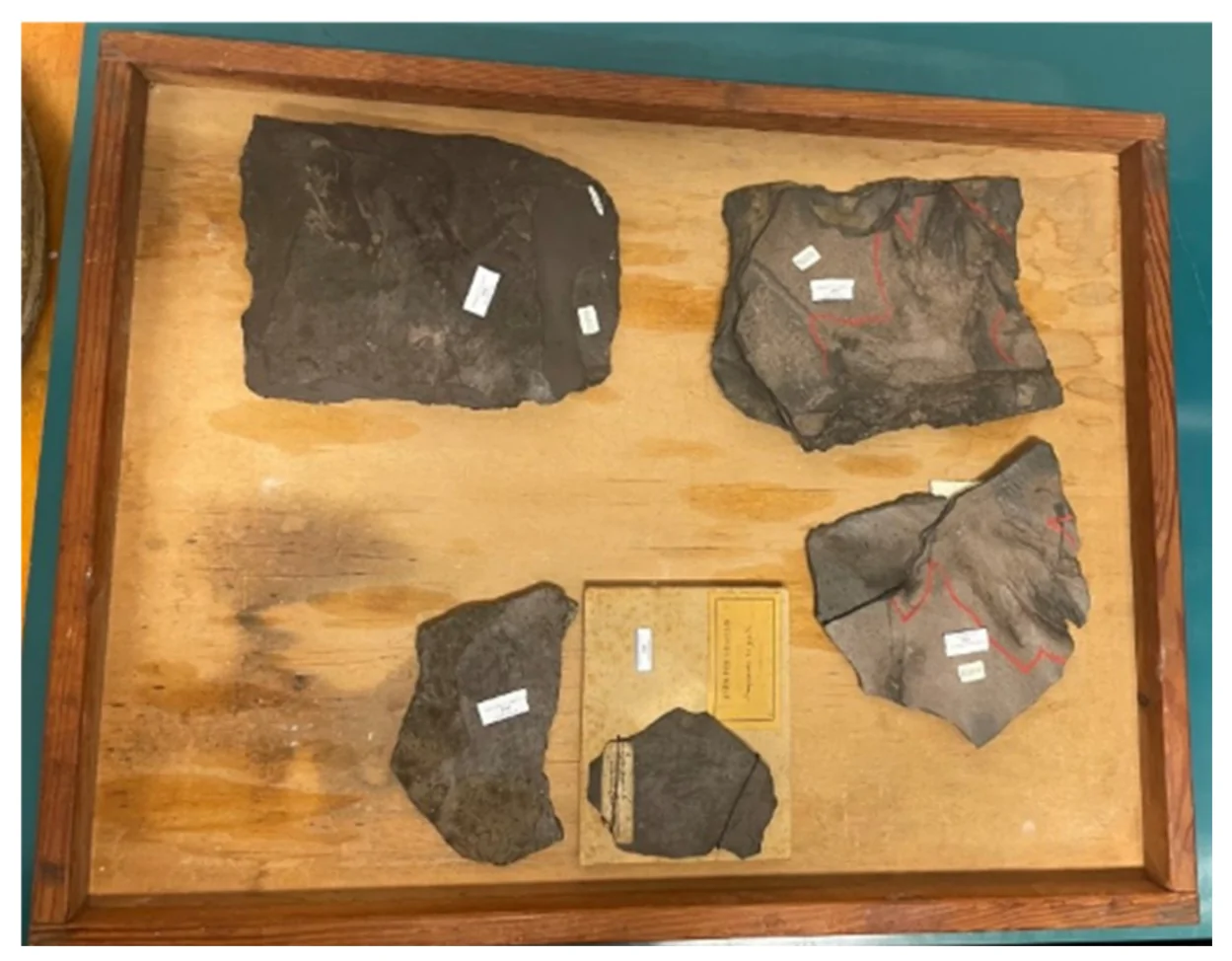
Dinosaur footprint from the “Giovanni Capellini Museum” Geological Collection.

**Figure 3:**
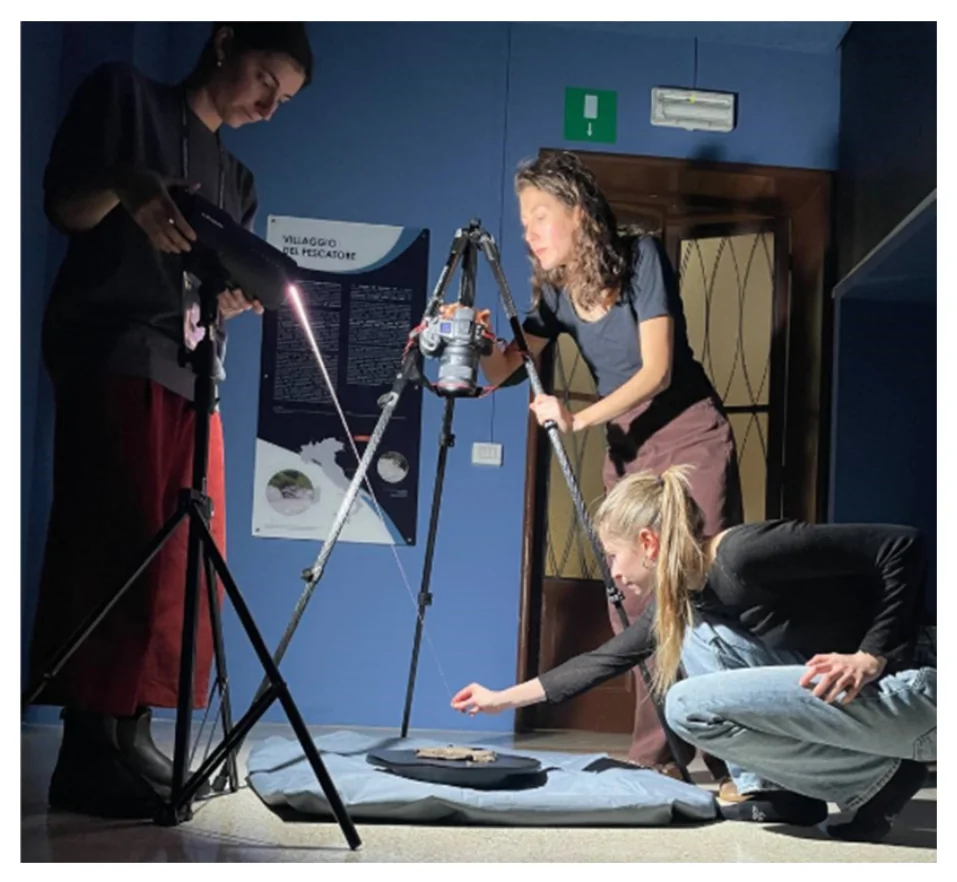
RTI acquisition process.

The RTI acquisition was conducted within the museum galleries and required the coordinated involvement of three operators, in addition to the collaboration of the museum curator. The RTI procedure was performed on the footprints using the Hasselblad X2D 100C (Hasselblad, Gothenburg, Sweden) equipped with a 38 mm lens (f/8, 1/30 s, ISO 400, −1.4 exposure compensation), without flash, in a completely dark environment. Images were recorded in RAW + JPEG format. Illumination was provided by a Profoto B10X Plus (Profoto, Stockholm, Sweden), which was manually moved along a circular trajectory around the object at three different heights, while maintaining a constant distance. The footprints Figure 2 (catalogue numbers: MGGC CNA1943, CNA1944, and CNA1946) were positioned on a rotating platform against a neutral grey background. Two reflective black spheres were fixed adjacent to the object to enable relighting calculations. Due to the lack of a dome system, the light source had to be manually displaced at three distinct elevations around the object. To ensure a consistent and proportional distance between the light and the subject, a measuring cord attached to the lighting unit was used to maintain a fixed radius (Figure 3).

For each footprint, a dataset of approximately 40 images was acquired. However, during the acquisition of two of the three footprints, minor camera micro-movements occurred, making part of the dataset unusable and reducing the number of valid images to approximately 24 per footprint. An additional factor that negatively affected the surface normal calculation was the use of a matte grey background, which introduced visual interference and contrast issues with the object’s cast shadows during post-processing. The resulting RAW files had dimensions of 11,656 × 8742 px and required compression to be imported and processed in relighting software.

**Figure 4:**
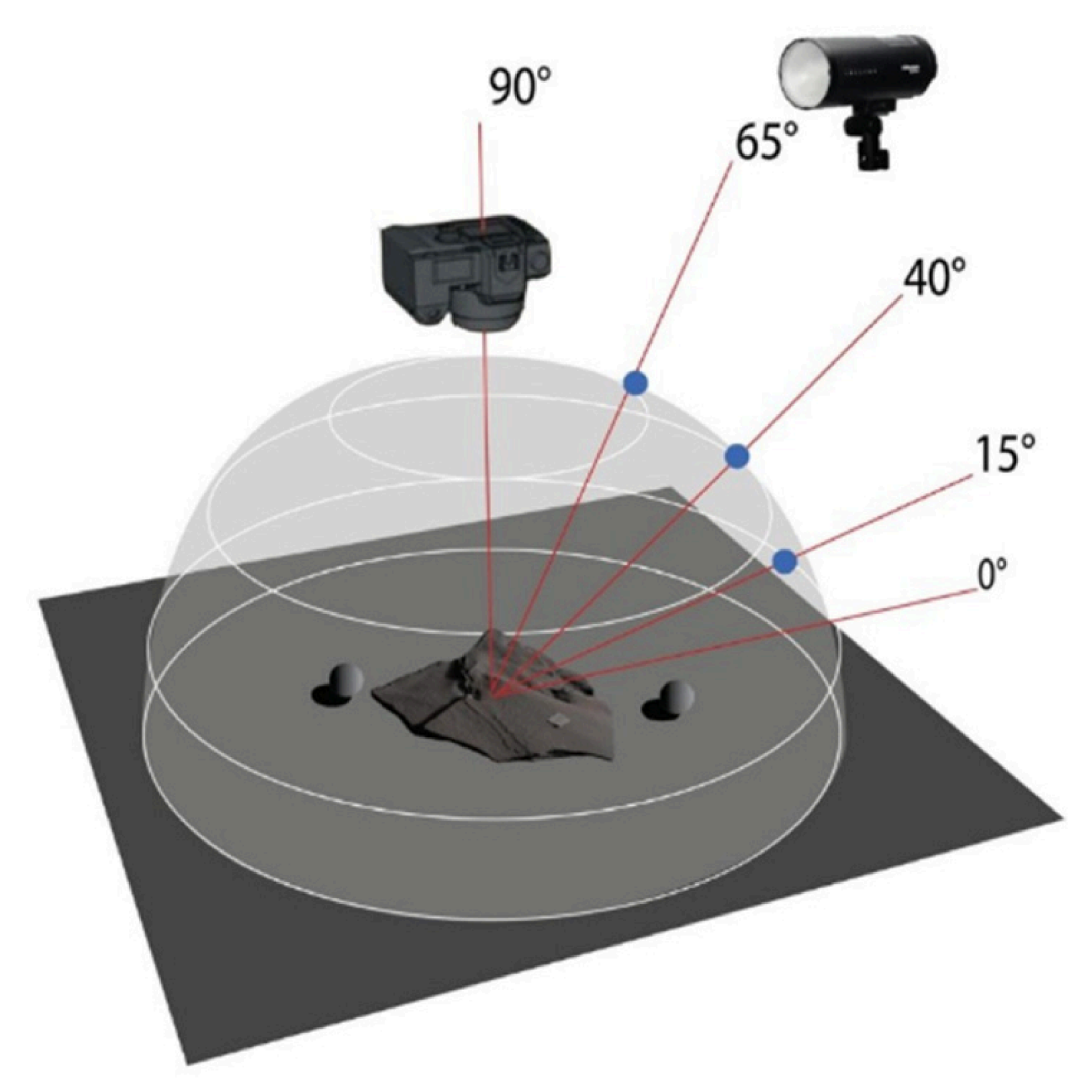
RTI acquisition scheme.

A second acquisition, aimed at obtaining a more accurate and precise dataset, was, therefore, carried out for one of the three footprints n. CNA1943, on which the assessment procedures were subsequently performed. This session employed a Canon EOS R3 (Canon Inc., Tokyo, Japan) with a 50 mm focal length lens (f/5.6, 1/60 s, ISO 640), again in a completely dark environment. The pipeline was otherwise identical to that previously described, with the exception of the use of a matte black background; the footprint was placed on a rotating platform, while the light was manually moved around the object at three different heights (93 cm, 53 cm, and 30 cm), for a total of 120 images (Figure 4). Since a different camera was used this time, the resulting RAW images had a resolution of 6032 × 4032 px, sufficient for the scope of this study and better suited for hardware with lower processing power. The images were then imported into the Adobe Camera Raw (version 2025) software for white balance calibration.

### 3.3. Three-Dimensional Scanning and Virtual Dome: A Standardized Workspace for VRTI Workflow

#### 3.3.1. Three-Dimensional Acquisition Campaign

To obtain a morphologically accurate three-dimensional model of the three artefacts, an Artec Space Spider II (Arted 3D, Luxemburg) was employed, supported by a turntable. The limited dimensions of the specimens, combined with the use of the round table, enabled a rapid and efficient acquisition of both sides of each artefact, while minimizing occlusions and discontinuities between scans. Data were captured according to the parameters detailed in Table 1. During post-processing in Artec Studio 19, the scans underwent preliminary cleaning and alignment, with removal of high-deviation frames and exclusion of the few scans exceeding a 0.2 mm global error or considered substantially compromised to ensure the reliability of the geometric dataset. From the selected scans, highly dense meshes were generated through fusion (Table 2). Minor gap-filling operations were required, primarily in small recesses and undercuts located along the margins of the artefacts, which were not fully accessible to the scanner due to geometric constraints. These semi-automatic filling procedures concerned exclusively peripheral areas that fall outside the paleontologist’s primary object of interest, namely, the footprint. The footprint itself was acquired and processed in its entirety, maintaining full morphological coherence with the original surface and without any loss of geometric information. As discussed in ref. [25], the adopted methodology is grounded in the preservation of multiple versions of the 3D model, differentiated according to their intended use. In the present case, where the dataset supports not only visualization but also scientific analysis, geometrically dense models were deliberately retained, prioritizing morphological resolution and completeness over optimization for real-time applications. The resulting 3D model constitutes a robust documentary resource, suitable for metric analyses, metadata production and technical documentation, as well as for conservation purposes and long-term deterioration monitoring.

#### 3.3.2. Virtual Dome Creation and Parameters Configuration

Following the generation of the 3D model, the VRTI was performed using the Virtual Dome (VD) specifically created to simulate RTI conditions [26] and generate the dataset required for the subsequent relighting phase (see Section 3.4). The VD was developed in the Blender 4.2 software. The dome comprised 60 virtual light sources arranged in three horizontal rings at varying heights and diameters, replicating the geometric distribution of the physical RTI dome. Each light was oriented between 40° and 65° relative to the surface, ensuring a consistent and sufficiently wide angular distribution to enhance the visibility of subtle morphological features during the virtual relighting process. Adjacent to the object, two black reference spheres (roughness value: 0.1) were positioned to enable accurate tracking of light vectors during subsequent processing. A fixed virtual camera was positioned at the top of the dome. Preliminary configurations explored different focal lengths, and multiple comparative tests of the rendering parameters using the Cycles rendering engine led to the identification of the most suitable configuration in terms of stability, visual clarity, and methodological coherence [26]. In the final workspace, therefore, the virtual camera was configured with a 50 mm focal length, a 36 × 24 mm sensor, and an output resolution of 8K px for rendering. These parameters have been embedded within a standardized and shareable Blender workspace, with the aim of ensuring consistency across acquisitions and facilitating reproducibility of the virtual RTI protocol. Finally, a custom script was developed with the support of the LLM ChatGPT 5.3 (https://chatgpt.com/, accessed on 1 July 2026) and integrated within the Blender workspace to automate the rendering process. From a methodological perspective, the workflow requires the following: the import and centering of the 3D model (origin set to geometry; coordinates x = 0, y = 0, and z = 0); alignment of the reference spheres along the X- and Y-axes on the same Z-plane as the object; proportional scaling of the predefined light groups (lights1, lights2, and lights3) according to the object’s dimensions; and maintenance of a camera orientation perpendicular to the local surface of the object of interest. The camera focal length (default: 50 mm), depth of field (focus set on the object), and output resolution (default: 8K) are explicitly defined prior to execution but can be modified accordingly with the object of interest, the hardware processing power, and the needed output. Within the script, the output file name and the directory should be manually specified to ensure controlled data storage and operating system consistency. The rendering logic is based on sequential activation of individual light sources: for each render, a single light is enabled, while all others are automatically deactivated, thereby generating a complete image set in which each frame is illuminated by one source at a time. Light management relies on the hierarchical folder structure embedded in the Blender file, allowing systematic identification and control of illumination groups. 

In the present context of a methodological comparison between VRTI and RTI, based on footprint CNA1943, strict spatial consistency was enforced within the Blender workspace by constraining the 3D model and virtual reference spheres to replicate the exact configuration of the RTI setup reference image. The RTI reference image was imported into Blender to guide the rotation and positioning of the model, ensuring perfect alignment in pixel space and spatial registration, maintaining geometric coherence between the physical and virtual datasets. After completing both the RTI and VRTI acquisition processes, the resulting data consisted, respectively, of photographic images and rendered images exported in JPG format with a resolution of 6032 × 4032 pixels. For each impression and for each technique, the two datasets were subsequently imported into the Relight 2024 version (https://vcg.isti.cnr.it/vcgtools/relight//, accessed on 15 May 2026). The same processing workflow, described in paragraph 3.4, was systematically applied to both RTI and VRTI datasets to eliminate analytical bias and ensure a controlled and reproducible comparison between the two methodologies.

### 3.4. Relighting: RTI and VRTI

**Figure 5:**
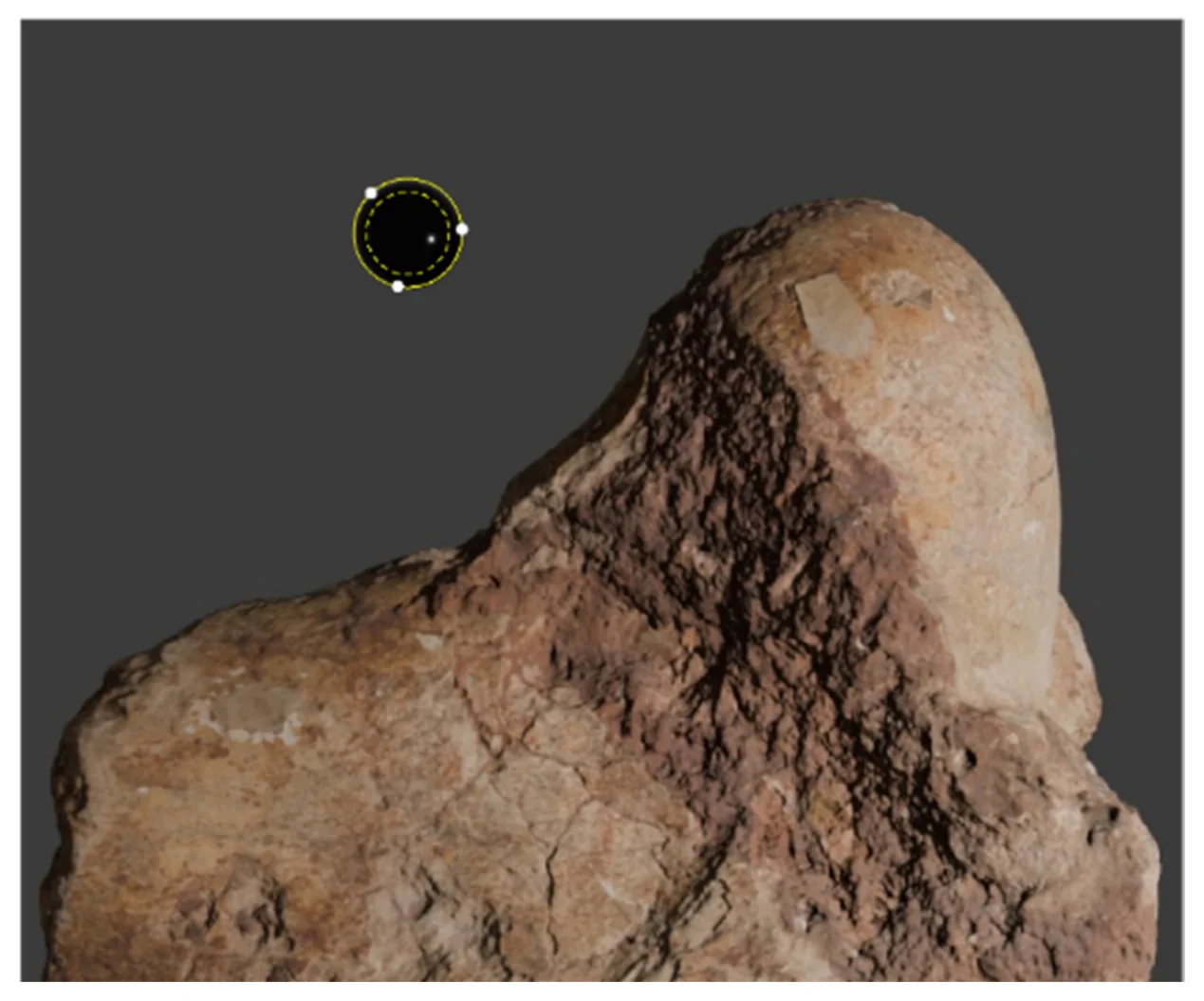
RTI acquisition scheme.

The processing phase consists of generating a .ptm file (Polynomial Texture Map), a reflectance representation format in which per-pixel luminance variation is modeled through polynomial functions of the incident light direction. This format enables interactive relighting of the surface, allowing users to simulate dynamic illumination conditions and thereby enhance the perception of fine morphological features. Prior to processing, the dataset undergoes a selection phase in which RAW images are reviewed, and any out-of-focus captures are excluded. The remaining RAW files are subsequently imported into the Relight 2024 software environment (Figure 5). The initial operation involves manually selecting the two reflective spheres visible in the first image; these spheres are then automatically detected across the remaining image set.

This step is critical for assessing the success of the RTI acquisition process, as even minimal micro-movements may alter the position of the spheres, resulting in images unsuitable for accurate relighting reconstruction. This limitation does not apply to VRTI rendering results, which are obtained within a fully virtual and controlled environment. When the sphere is selected in all the images, the operator selects the light source reflected on the sphere in each image, now automatically enabled in the Relight 2025 version. Finally, the .ptm file is computed.

### 3.5. User-Based Evaluation on Footprint Readability

**Figure 6:**
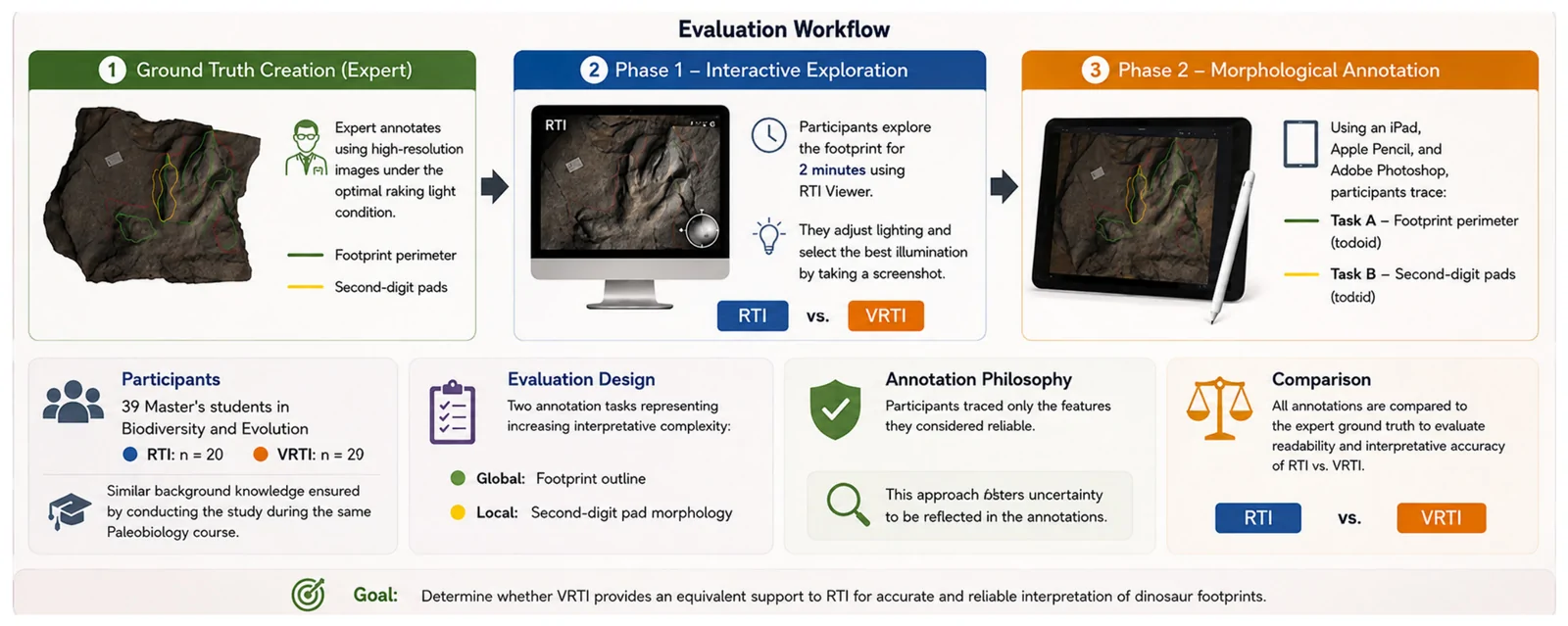
The comparative testing protocol and user evaluation pipeline.

Given the primary aim of this study, namely, the development of a tool to support domain experts in the interpretation of paleontological footprints, comparative assessment of footprint readability was conducted between RTI and VRTI (Figure 6). The resulting evaluation protocol was subsequently published on Protocols.io [27]. The aim of the assessment is to quantitatively evaluate whether RTI and VRTI differ in their ability to support the interpretation and delineation of morphological features by paleontology experts and scholars. Footprint identification in paleontology typically relies on the delineation (often manual) of the footprint perimeter and the identification of morphological features such as fingers, palms and digit pads. Accordingly, the present assessment was designed to replicate, as closely as possible, the operational conditions and analytical workflow of a professional paleontologist. A total of 39 users (RTI: *n* = 20; VRTI: *n* = 19) were asked to manually delineate selected features of fossilized dinosaur footprints in the CNA1943 case study. After an initial exploration phase using an RTI viewer, where they were asked to identify the coordinates of the best light perspective in their opinion to observe morphological features, participants traced contours on a tablet device (iPad) using Adobe Photoshop 2025 (https://www.adobe.com/products/photoshop.html, accessed on 15 April 2026). Two tasks were designed to represent two levels of increasing interpretative complexity:

A. Global contour: Outline of the footprint;
B. Local detail: Contour of a finer anatomical feature of the second digit (phalanges).

Participants were explicitly instructed to trace only those regions they considered reliable, allowing omissions to reflect interpretative uncertainty. 

#### 3.5.1. Materials

**Figure 7:**
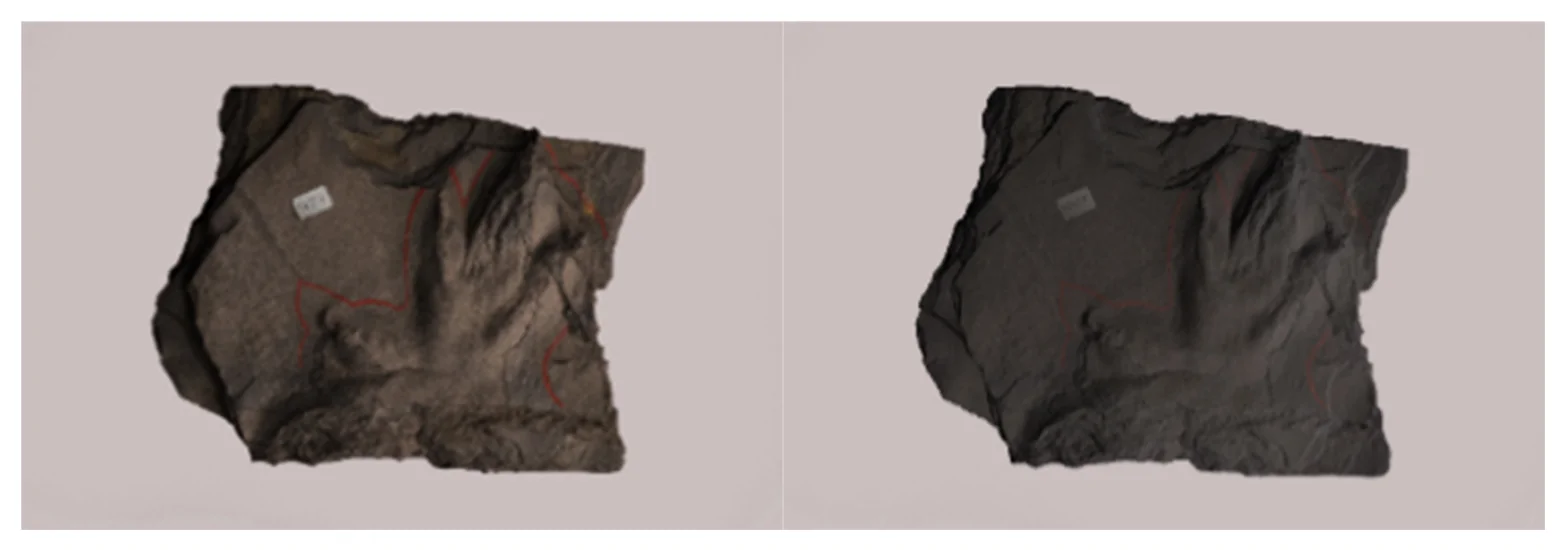
High-resolution RTI and VRTI footprint snapshots under raking-light condition.

The ground truth was established through expert annotation. For each RTI and VRTI file in .ptm format, two high-resolution snapshots (6000 × 4000 px) were captured from the RTI Viewer. These images were subsequently imported into Adobe Photoshop, spatially aligned, and re-exported at a resolution of 3240 × 2160 pixels under two lighting conditions: (i) diffuse illumination and (ii) raking light (x = 0.71; y = −0.37). A domain expert was asked to evaluate the two lighting conditions and select the one most suitable for morphological interpretation aimed at identifying the footprint perimeter and digital pad features. The raking-light condition was selected for subsequent annotation (Figure 7).

**Figure 8:**
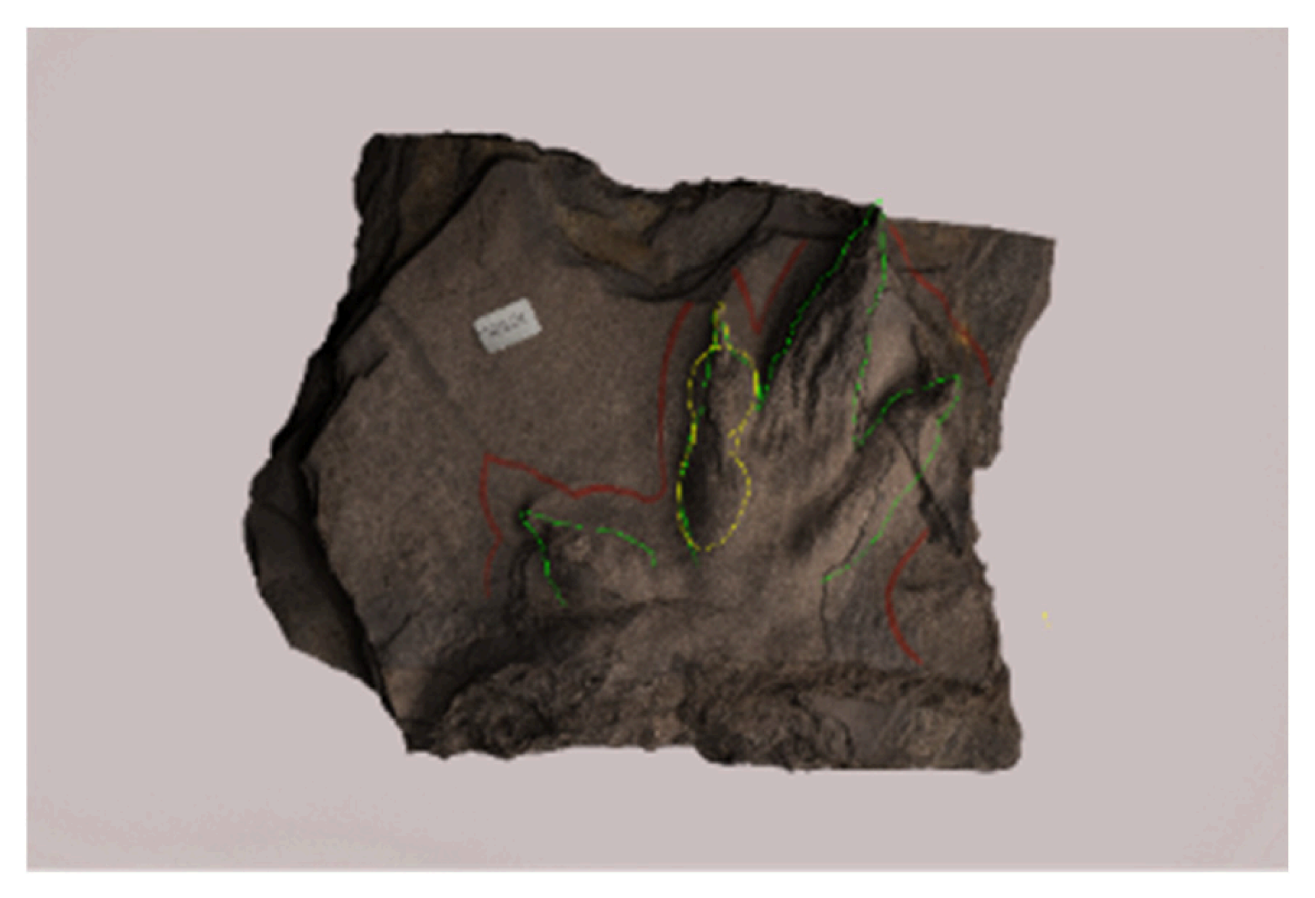
Ground-truth data: [A.] footprint perimeter (green) and [B.] local detail (yellow).

To ensure consistency, particular attention was given to the precise spatial correspondence and resolution matching the generated images, allowing annotations to be directly overlaid without geometric discrepancies. The selected image was then used by the expert to define the ground truth, annotating it in Adobe Photoshop using two separate layers (Figure 8): one for the footprint perimeter [a] and one for the pads of the second digit [b]. Tracing was carried out on an iPad using an Apple Pencil, with standardized and absolute color coding:

A. Green for the footprint perimeter with RGB value (0, 255, 0);
B. Yellow for the digit pad with RGB value (255, 255. 0).

#### 3.5.2. Participants and Procedure

**Figure 9:**
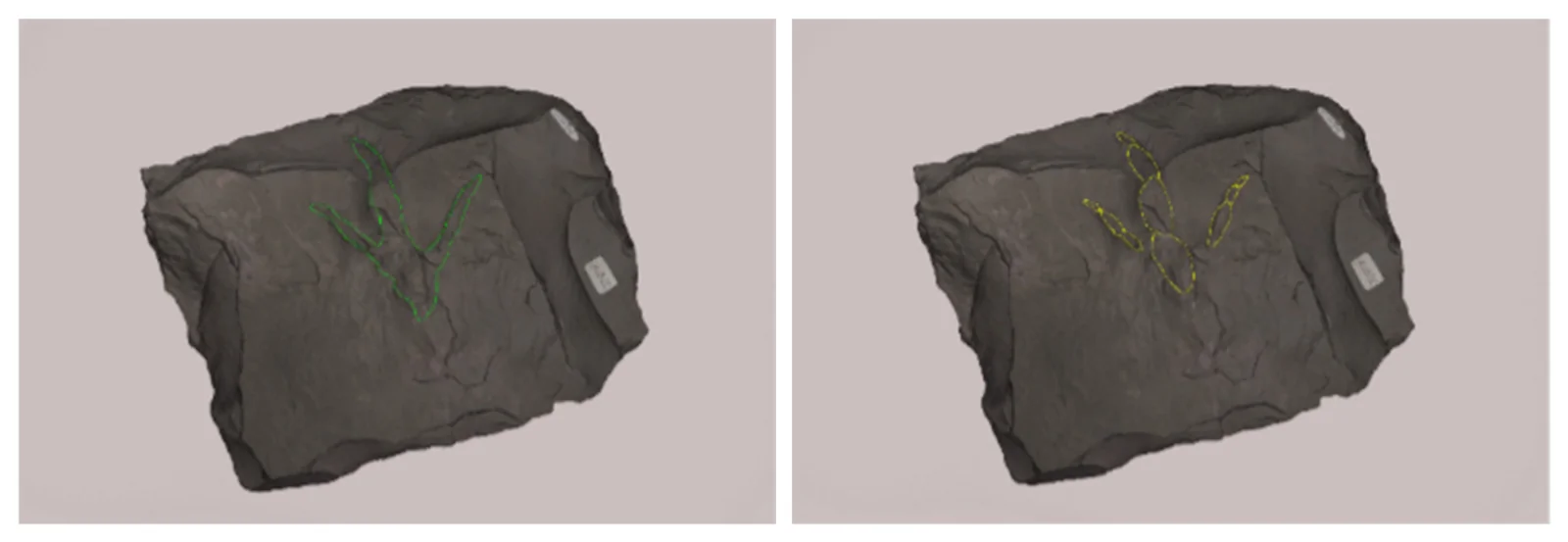
Example of CAN 1946 footprint contour (**left**) and digit pad identification (**right**).

To ensure high cohort homogeneity and eliminate domain-knowledge disparities, the participant selection and testing schedule were established in direct coordination with the course professor. Following the ground-truth definition, a cohort of 39 fourth-year master’s students in biodiversity and evolution attending the paleobiology course participated in this study. Participants were split randomly into two parallel groups, RTI (*n* = 20) and VRTI (*n* = 19), based on their seating arrangement in the classroom during scheduled lecture hours. The evaluation was deliberately conducted at the exact midpoint of the course, ensuring that all participants shared a comparable educational baseline regarding paleontological footprint morphology prior to testing. Furthermore, the digital tasks were specifically designed to be highly intuitive, thereby minimizing technical software barriers and reducing the likelihood that prior experience with advanced 3D or RTI software would act as a primary confounding factor. Before starting the testing, an illustrative image of the same procedure carried out on a different footprint, CNA1946, was shown (Figure 9).

The assessment consisted of two sequential phases carried out by both groups in parallel in accordance with the specific methodology assigned to them.

**Phase 1: Interactive Exploration and Lighting Selection**

**Figure 10:**
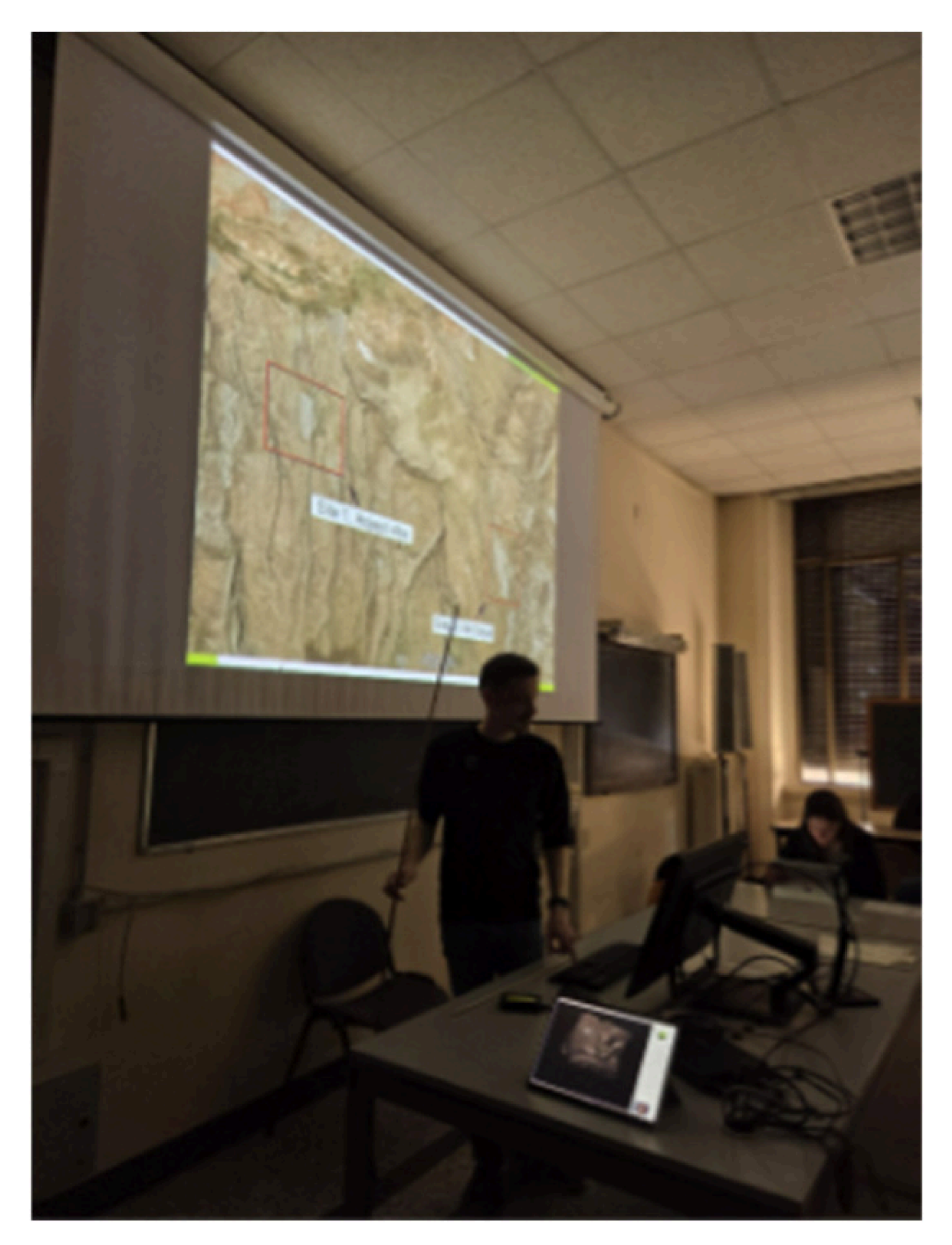
Interactive exploration and lighting selection, phase 1.

**Figure 11:**
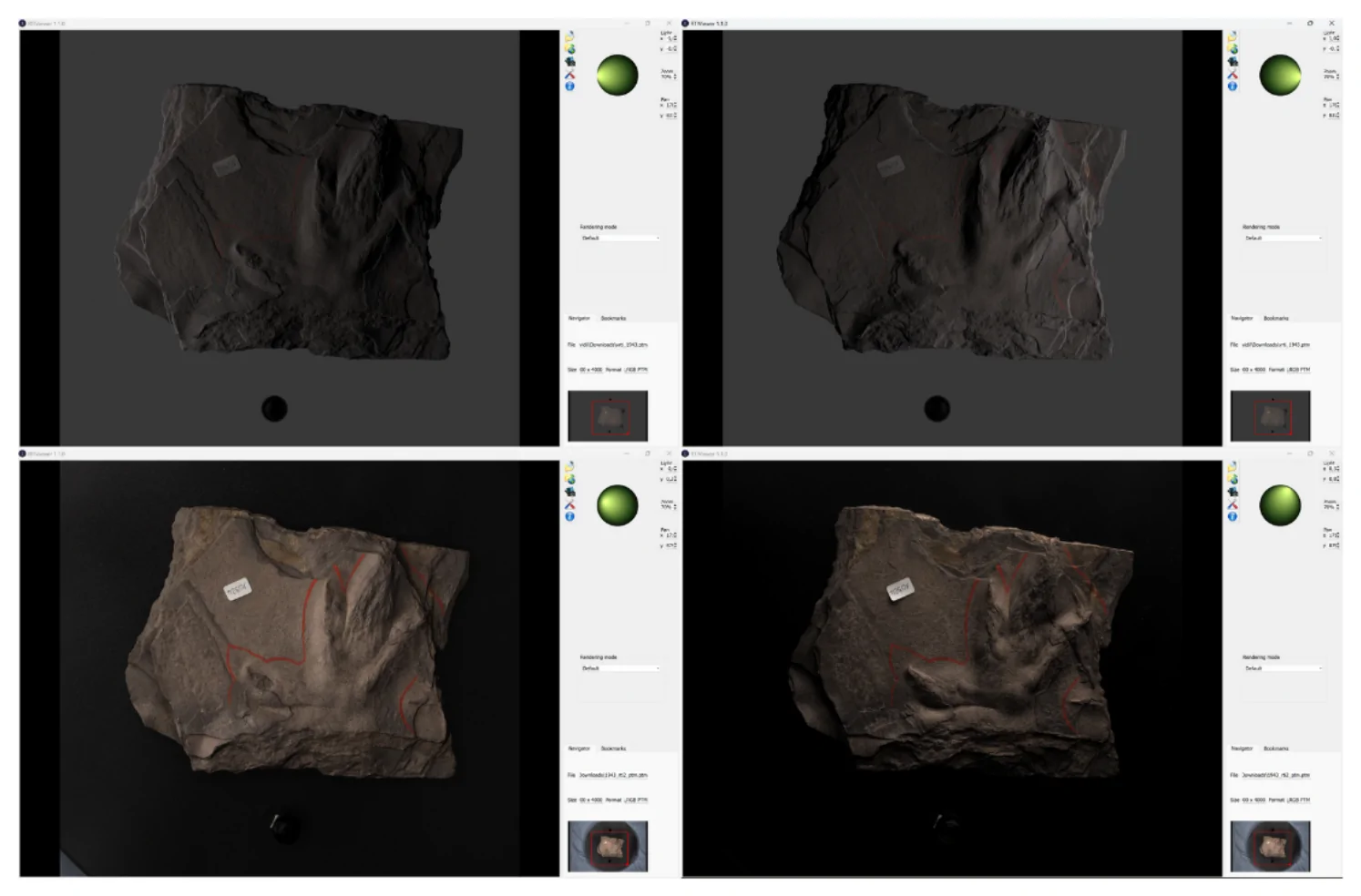
Light perspective examples of RTI (**bottom**) and VRTI (**top**).

Participants were individually provided with a tablet running RTI Viewer and given 2 min to explore the footprint morphology by interactively adjusting the direction of the lighting (Figure 10). At the end of the exploration period, participants were instructed to capture a screenshot representing the lighting configuration they considered most suitable for morphological interpretation (Figure 11).

**Phase 2: Morphological Annotation**

Participants, divided into RTI and VRTI groups, were then moved to a separate workstation equipped with an iPad and Apple Pencil. A pre-configured Adobe Photoshop file was provided, containing the footprint image on a neutral background and a set of transparent annotation layers. The number of layers corresponded to twice the number of participants, organized in pairs corresponding to (A) perimeter and (B) digit pad annotation.

**Figure 12:**
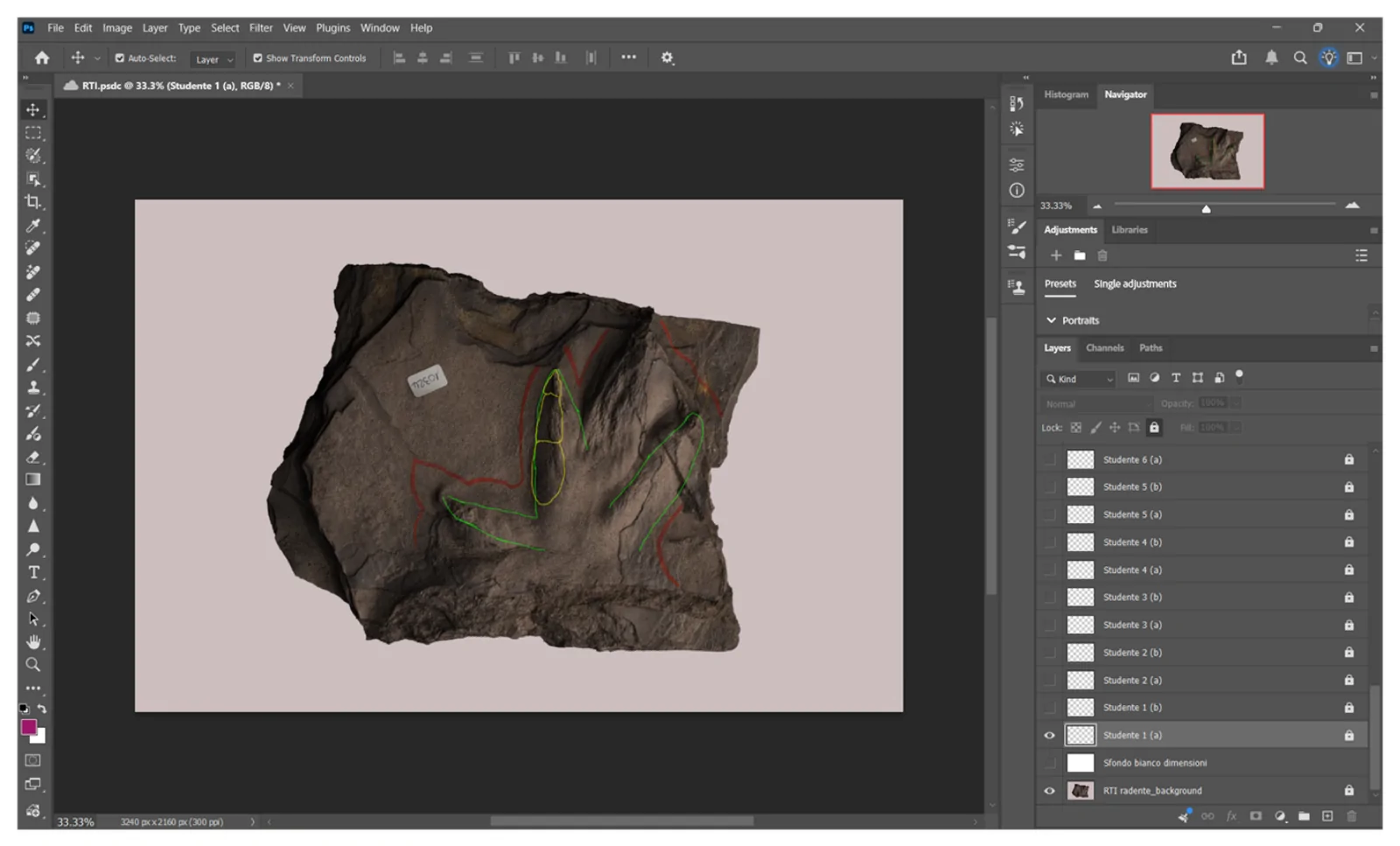
The test workspace in Adobe Photoshop.

Each participant was assigned a unique pair of layers and instructed to replicate the expert annotation procedure: tracing the footprint perimeter on layer (A) using a green brush tool and marking the pads of the second digit on layer (B) using a yellow one (Figure 12).

#### 3.5.3. Evaluation Framework

The following section outlines the analytical framework adopted to evaluate the results, combining both quantitative and qualitative approaches. To ensure transparency and reproducibility, a GitHub repository was specifically created for this project (https://github.com/VIDILAB-UNIBO/VRTIandRTI_project/blob/main/VRTIvalidation_workflow_notebook.ipynb, accessed on 27 August 2026). This repository contains a Jupyter Notebook v6.4.6 (Python 3.7.9) detailing the full analysis workflow step by step, including all scripts used and the resulting datasets. In the interest of full disclosure, it is noted that large language models (LLMs) were used as collaborative tools to support the development and optimization of the Python scripts provided in the analysis.

Three complementary evaluation strategies were employed:

1. Quantitative analysis, based on the comparison between contours traced by participants and the expert-defined ground truth (in both Tasks A, a global footprint outline, and B, a local anatomical detail). This comparison was implemented through a custom Python pipeline, enabling data analysis and statistical assessment of the comparability between RTI and VRTI as interpretative support tools.
2. Qualitative analysis, based on expert evaluation, was designed as a complementary descriptive index to contextualize the primary quantitative spatial metrics. The paleontologist who defined the ground truth reviewed the outputs using a 5-point Likert-type ordinal scale, providing domain-specific insights and interpretative commentary. This qualitative layer supports the quantitative findings by contextualizing the observed patterns and highlighting relevant morphological considerations. The analysis of the qualitative scores given by the expert to the results has been included in the same Jupyter Notebook to guarantee the completeness of the data obtained.
3. Finally, the preferred lighting perspective selected by participants was analyzed as a secondary integrative layer. By recording the specific light source coordinates (X, Y) chosen to best highlight morphological features, visual perception and subjective interpretation were evaluated across both mediums through statistically significant assessment.

**a.**  **Quantitative analysis—data preparation and processing**

**Figure 13:**
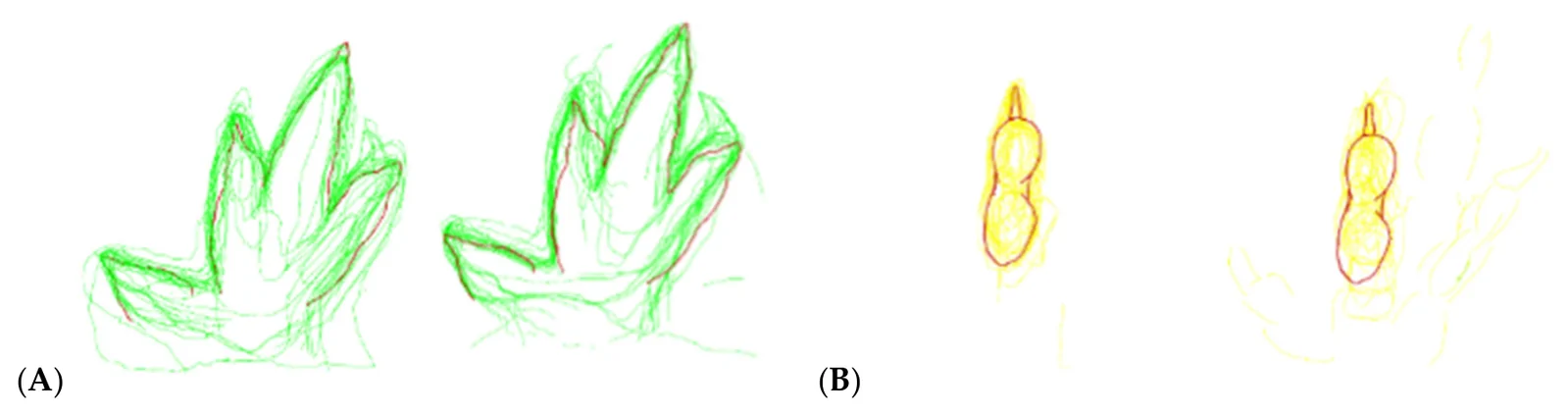
Footprint and digit pad tracings for RTI (**A**) and VRTI (**B**) groups. Ground truth is in red for visualization purposes.

For what concerns the quantitative analysis approach, first, all contours were exported as PNG images (Figure 13) and processed using a custom Python pipeline. Manually traced contours were isolated based on predefined RGB color values (green for Task A and yellow for Task B) and subsequently converted into spatial data, represented as 2D point sets stored in XYZ format. Each point was defined within a Cartesian coordinate system, where pixel positions were mapped onto the X- and Y-axes, while the Z-coordinate was set to zero.

Following the conversion of raster images into spatial coordinates, a grid-based subsampling [28] was applied to all generated point clouds. This step was necessary to normalize point density across datasets. Subsampling ensures that variations in contour length or thickness do not introduce bias into distance-based metrics such as RMSE. In particular, it prevents longer or denser segments from disproportionately influencing the results. Additionally, it mitigates noise introduced by anti-aliasing effects, ensuring that the statistical comparison reflects geometric accuracy rather than rendering artifacts.

**Distance computation**

Since students were instructed to delineate only the morphological features they deemed most reliable, two complementary analytical approaches to evaluate their performance were adopted. Both methods rely on cKDTree, a space-partitioning data structure that allows for high-speed nearest neighbor (NN) searches [29]. By organizing the point clouds into a binary tree, the algorithm efficiently identifies the minimum distance between any point in the student’s dataset and the corresponding ground truth.

- Unidirectional distance (primary metric)

The primary metric is a unidirectional analysis that calculates the distance from each point in the student’s tracing to the nearest point in the ground truth. This approach is highly consistent with our testing protocol, as it evaluates the accuracy of the traced regions without penalizing omissions. In this context, omitted segments were considered indicators of interpretative uncertainty rather than errors; thus, this metric focuses strictly on the precision of the data provided.

- Bidirectional Chamfer distance (complementary analysis)

As a secondary analysis, a bidirectional (Chamfer) distance was computed [30]. This metric incorporates both local accuracy and contour completeness by calculating distances in both directions: from student to ground truth and vice versa. This enables the assessment of omitted regions, providing a broader overview of how much of the original footprint was successfully identified and traced. 

The combined use of unidirectional and bidirectional distance measures allows for a more nuanced interpretation of participant performance. While the unidirectional metric captures local geometric accuracy, the bidirectional metric highlights differences in contour completeness. This distinction can be potentially relevant in contexts where users are encouraged to selectively interpret uncertain features, as it enables the separation of perceptual accuracy from the overall interpretative strategy of the participants. While the current paper details the results of the unidirectional analysis, the corresponding Jupyter Notebook includes the bidirectional script block (provided as commented code). This allows for future reuse and verification, enabling researchers to switch metrics if their objectives prioritize contour completeness over interpretative precision. Furthermore, both the unidirectional and bidirectional CSV datasets have been made available in the project repository.

**Statistical analysis and visualization**

Distance distributions were summarized using the following metrics:

- **Root-mean-square error (RMSE):** Used as the primary metric, as it preserves the original measurement units (pixels) while penalizing larger deviations, providing a robust measure of overall geometric accuracy.
- **Ninetieth percentile (P90):** Calculated to identify the error threshold for 90% of the tracing, offering a reliable representation of typical performance by reducing the influence of extreme outliers.
- **Standard deviation (StdDev):** Used to assess the consistency and variability of the tracing stroke across each participant’s dataset.

The maximum distance (Max) and simple mean distance were excluded from the final analysis to avoid sensitivity to isolated input errors (outliers) and to focus on more statistically significant indicators of performance. 

Statistical comparisons between RTI and VRTI conditions were performed separately for each task (A and B). Prior to the analysis, the normality of the distributions was assessed using the Shapiro–Wilk test [31]. The test revealed that the primary metric (RMSE) for Task A deviated significantly from normality (*p* < 0.05). Consequently, a non-parametric Mann–Whitney U test [32] was employed to assess differences between two independent samples (RTI and VRTI). This test was maintained across all comparisons, including those where normality was not strictly violated, to ensure methodological consistency and to provide a robust, conservative estimate, given the relatively small sample size (n ≈ 20). Following common practice, a significance threshold of (*p* < 0.05) was adopted. To complement the null-hypothesis significance testing and assess the practical magnitude of the differences between the two modalities, effect sizes were calculated for the Mann–Whitney U tests using the rank-biserial correlation coefficient ($r_{rb}$). Values of $|r_{rb}|<0.3$ were interpreted as small effect sizes, indicating practical consistency between the groups. Additional descriptive statistics (e.g., mean, max and standard deviation) were also computed for each metric to characterize the distribution of contour deviations, enabling a more complete overview within the published CSV datasets.

To compare RTI and VRTI conditions, RMSE values were visualized using boxplots combined with jittered scatter plots. This representation was chosen to provide a transparent view of the entire dataset, displaying medians, interquartile ranges, and individual participant distributions (see Figure 12 in Section 4.1). This approach is suitable for our sample size and aligns with the non-parametric statistical framework adopted, as it does not assume a normal distribution of the data.

**b.**  **Qualitative analysis—data preparation and evaluation approach**

To complement the quantitative metrics, a qualitative evaluation was conducted by the domain paleontologist who established the ground-truth annotations. The expert was asked to examine each student tracing, blind to the RTI/VRTI conditions. To evaluate interpretative quality and morphological readability, a 5-point Likert-type ordinal scale was developed specifically for this study, tailored directly to the diagnostic features of this fossil specimen. Its purpose was to provide a consistent way for the domain expert to evaluate the morphological and interpretative quality of each tracing with respect to the ground truth. The scale ranged from 1 to 5, corresponding to the following:

1. Outline very distant from the track morphology, including large missing parts;
2. Outline distant from track morphology, with minor missing parts;
3. Outline showing moderate correspondence with the track morphology, lacking digital pads outline;
4. Outline showing good correspondence with the track morphology, but lacking detailed pads outline;
5. Optimal outline, closely consistent with the track morphology, including digital pads.

The scale was selected to provide sufficient granularity to distinguish different levels of interpretative quality while avoiding an unnecessarily fine-grained assessment. To analyze these ordinal Likert ratings, the results are summarized using non-parametric distribution measures (median and interquartile range (IQR)) alongside descriptive parametric metrics (mean and standard error of the mean (SEM)) to provide a comprehensive qualitative assessment of tracing quality across experimental conditions.

**c.**  **Integrating interpretative data: lighting perspective**

As a secondary, integrative layer of the evaluation framework, this study provides an analysis of the lighting perspective selected by participants at the end of their exploration phase as best highlighting the morphological characteristics of the artifact.

The light coordinates were subsequently extracted from the captured screenshots and compiled into a numerical dataset for each participant and condition. Unlike the RMSE metric analysis, which measured execution accuracy relative to the ground truth, this study focuses on visual perception and subjective interpretation. The analysis aimed to identify patterns and thereby determine whether a shared “optimal light” consensus exists or if the choice is purely subjective. 

This study evaluated the impact of the visualization medium. It was tested whether the VRTI shifts or distorts the perception of relief compared with traditional RTI visualization. Consistent with the previous analysis, the normality of the lighting coordinate distribution was first verified using the Shapiro–Wilk test, which confirmed a non-normal distribution for several variables. Consequently, statistical significance was assessed using the Mann–Whitney U test, chosen for its robustness against outliers and its suitability for small, non-normally distributed datasets.

Finally, to further investigate whether individual lighting behaviors influenced interpretation performance, a Spearman rank correlation analysis was conducted between the lighting positions (computed as the Euclidean distance from the center of the light dome) and the final tracing accuracy (RMSE). The Spearman method was selected because it is a non-parametric test that evaluates monotonic relationships without assuming a normal distribution of the spatial coordinate data, while also offering robust resistance to potential outliers in user lighting selections.

### 3.6. Validation and Standardization of the Workspace

After evaluating the readability of the VRTI in comparison with the RTI, a usability evaluation of the virtual workspace developed in Blender was conducted. The primary objective of this assessment was to verify that the workspace ensured a high degree of scalability and adaptability across different types of 3D models, while effectively addressing the needs of cultural heritage professionals working with Blender and related 3D technologies. To this end, a heterogeneous group of participants was recruited, including professionals involved in cultural heritage digitization as well as users with at least a basic level of familiarity with Blender.

In the first round of testing, six responses were collected from domain professionals. Although this sample is insufficient to ensure statistical validity, it provided valuable preliminary feedback. Subsequently, the test was administered to a class of students enrolled in the second year of the Digital Humanities and Digital Knowledge master’s degree at the University of Bologna, attending the laboratory of 3D modeling and visualization in cultural heritage. Participants were provided with an overview of both the research project and the tool developed prior to testing.

The testing procedure required the following:

- Approximately 15 min to set up the workspace and launch the VRTI rendering process.
- Approximately 1 h for rendering, depending on hardware specifications and the selected output resolution. This phase did not require active user intervention and could be executed in the background.
- No more than 15 min to complete a final anonymous questionnaire.

The questionnaire incorporated the System Usability Scale (SUS), a validated ten-item instrument designed to assess perceived usability [33], with the intent of evaluating the usability of the workspace itself rather than the technical performance or expertise of the participants. Before completing the SUS, users were asked to generate a self-selected identification code that did not reveal their identity. Additional questions collected information regarding prior familiarity with the RTI technique and level of experience with Blender, enabling contextual interpretation of the results. Since SUS provides a global measure of perceived usability rather than a diagnostic assessment of individual workflow stages, an open-ended comments section was included to gather qualitative feedback and support a more comprehensive evaluation of the tool. Participants were also invited to share the dataset generated through their VRTI test via SwissTransfer (https://www.swisstransfer.com/it, accessed on 11 May 2026), attaching the folder labeled with their chosen ID. SwissTransfer was selected because it does not store the sender’s email address; the ID served exclusively to associate the submitted dataset with the corresponding questionnaire responses while ensuring full anonymity. In addition to its original functionalities, the script was further extended to automatically export the resulting images in .jpg format along with a .txt paradata file containing the following information: focal length, sensor fit, camera position (XYZ), render engine, resolution, Cycles sample count, and an exported image list (including each filename and the corresponding light name).

## 4. Results

### 4.1. Quantitative Results: Unidirectional Point Clouds Analysis 

**Figure 14:**
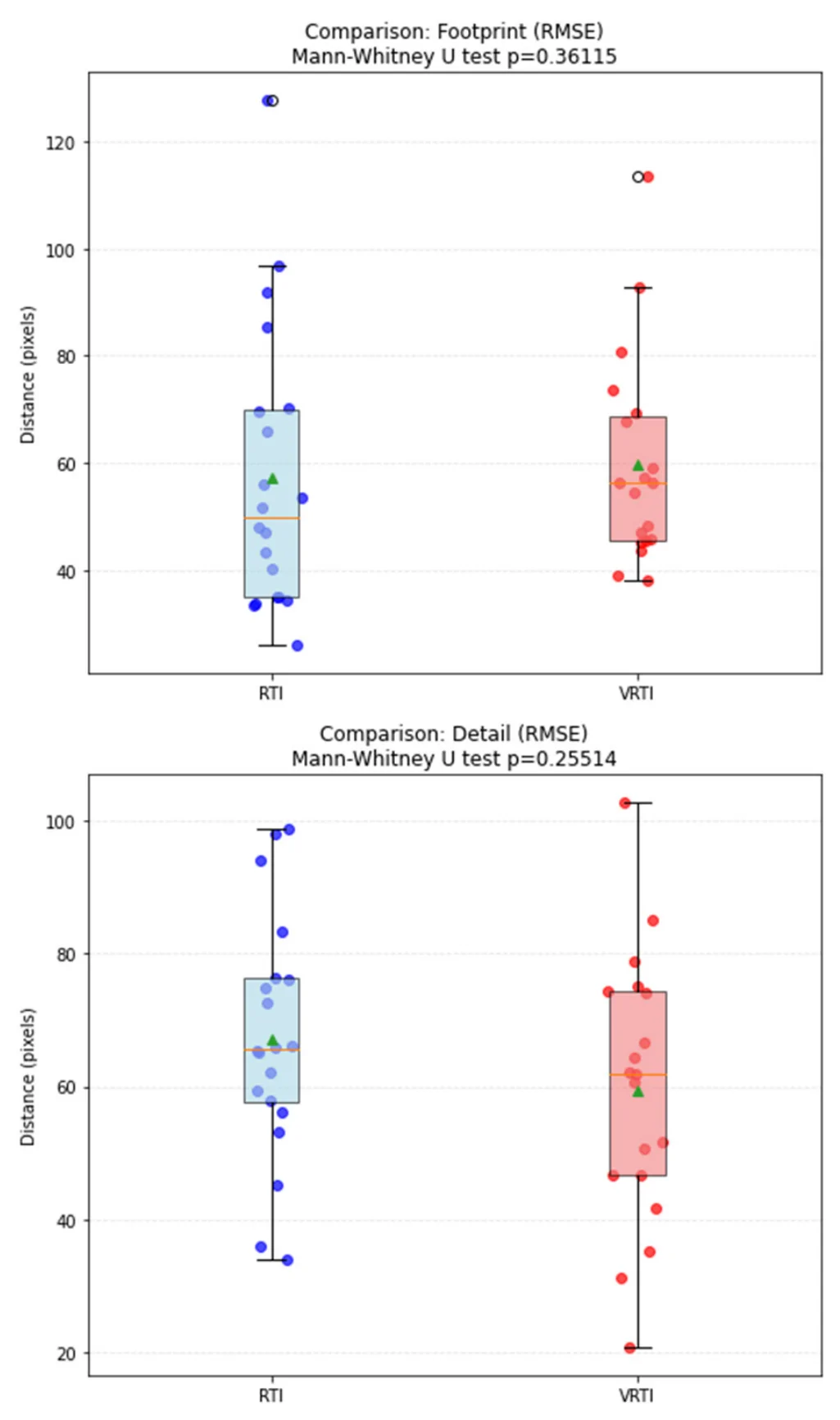
RMSE distribution for Tasks A and B. The boxplots display the medians (horizontal line), the interquartile ranges (box), and the means (green triangle) for both RTI and VRTI conditions. Distance is expressed in pixels.

The primary metric used to assess tracing accuracy was the unidirectional RMSE, which measures the distance from each point of the student’s tracing to the nearest point on the ground truth. The results obtained are summarized in Figure 14 and Table 3. This approach was chosen to avoid the penalization of intentional omissions. However, it is noteworthy that even when applying the complementary bidirectional metric, the statistical comparison between RTI and VRTI remained non-significant (*p* > 0.05) for both tasks. This consistency across different analytical approaches suggests that the choice of visualization medium did not introduce substantial systemic biases in either tracing accuracy or interpretative completeness within our sample.

Task A (Footprint): The analysis showed consistency between the two groups. The boxplot (Figure 12) illustrates the distribution of unidirectional RMSE values for the footprint-tracing task, comparing the RTI and VRTI groups. Both groups exhibit a similar spread of data, with medians (the orange lines) positioned between 50 and 60 pixels. The interquartile ranges (IQRs), represented by the heights of the boxes, are largely overlapping, indicating that the overall precision of the participants remained consistent regardless of the visualization medium.

The green triangles indicate the mean values (RTI: 57.30; VRTI: 59.70). The proximity of these means suggests that the VRTI did not introduce any significant bias or distortion in the perception of the general perimeter. The Mann–Whitney U test confirmed that there is no statistically significant difference between RTI and VRTI for the footprint-tracing task (U=223.0; p=0.361). Furthermore, the rank-biserial correlation yielded a small effect size ($r_{rb}=-0.174$), reinforcing the practical and perceptual consistency between the two visualization media. Finally, the scatter points (jitter) show a few individual outliers in both groups (points above 100 pixels), which likely represent students who encountered specific interpretative difficulties. However, the core of both samples is concentrated in the lower-error zone (35–70 pixels), suggesting a high level of “visualization medium transparency” for this specific task.

Task B (Detail): Interestingly, when removing the penalty for omitted regions, the performance gap narrows significantly. In fact, the complementary comparison with the bidirectional (Chamfer) metrics revealed significantly higher RMSE mean values for Task B (RTI: 187.52; VRTI: 188.45). This discrepancy highlights a specific behavioral pattern: while participants maintained high geometric accuracy in the strokes they produced (low unidirectional RMSE), they frequently omitted complex segments of the detail. As shown by the boxplot (Figure 14), the detail tracing (Task B) reveals a higher degree of individual variability compared with the footprint (Task A), reflecting the increased complexity of interpreting fine morphological features. In this task, the VRTI group achieved a lower mean RMSE (59.46) compared with the RTI group (67.04), as indicated by the green triangles. Despite the lower mean in VRTI, the Mann–Whitney U test confirms that the difference between the two modalities was not statistically significant (U=149.0; *p* = 0.25514). The calculated rank-biserial correlation effect size was also small ($r_{rb}=0.216$). The interquartile ranges (IQRs) of both groups show a substantial overlap, which, combined with the small effect size, reinforces the conclusion that both RTI and VRTI show comparable performance for high-detail documentation. 

The vertical spread of the data points (jitter) is more pronounced in these task results. In the VRTI group, a wider range of performance was observed, with some participants achieving exceptionally low error rates (around 20–30 pixels), while others reached over 100 pixels. Notably, unlike Task A, these higher values in Task B fall within the upper whisker boundary and do not represent statistical outliers. This suggests that, despite the comparable performance of VRTI and RTI, individual interpretative variability remains particularly relevant when dealing with complex morphological details. Given that the errors are measured in pixels within a normalized Cartesian coordinate space, a mean distance of approximately 60 pixels across both groups represents a high level of precision for a manual tracing task. Specifically, all exported contours were processed at a uniform high 3240×2160 pixels (3.5.1), where one pixel corresponds to approximately 0.1 mm on the physical specimen surface. Consequently, a mean unidirectional RMSE of ~60 pixels translates to a physical deviation of roughly 6 mm. Relative to the overall dimensions of the fossil footprint (approx. 30–35 cm), a physical error margin of ~6 mm represents a minor relative error (less than 2% of the specimen length). Within the scope of this study, this level of variation reflects a consistent degree of spatial agreement for manual freehand tracing performed by non-expert annotators, supporting its practical utility for qualitative morphological documentation. The transition from Task A to Task B did not significantly increase the RMSE when using the unidirectional metric, indicating that students maintained their spatial accuracy even when the subject became more difficult.

### 4.2. Qualitative Results: Expert Evaluation 

**Figure 15:**
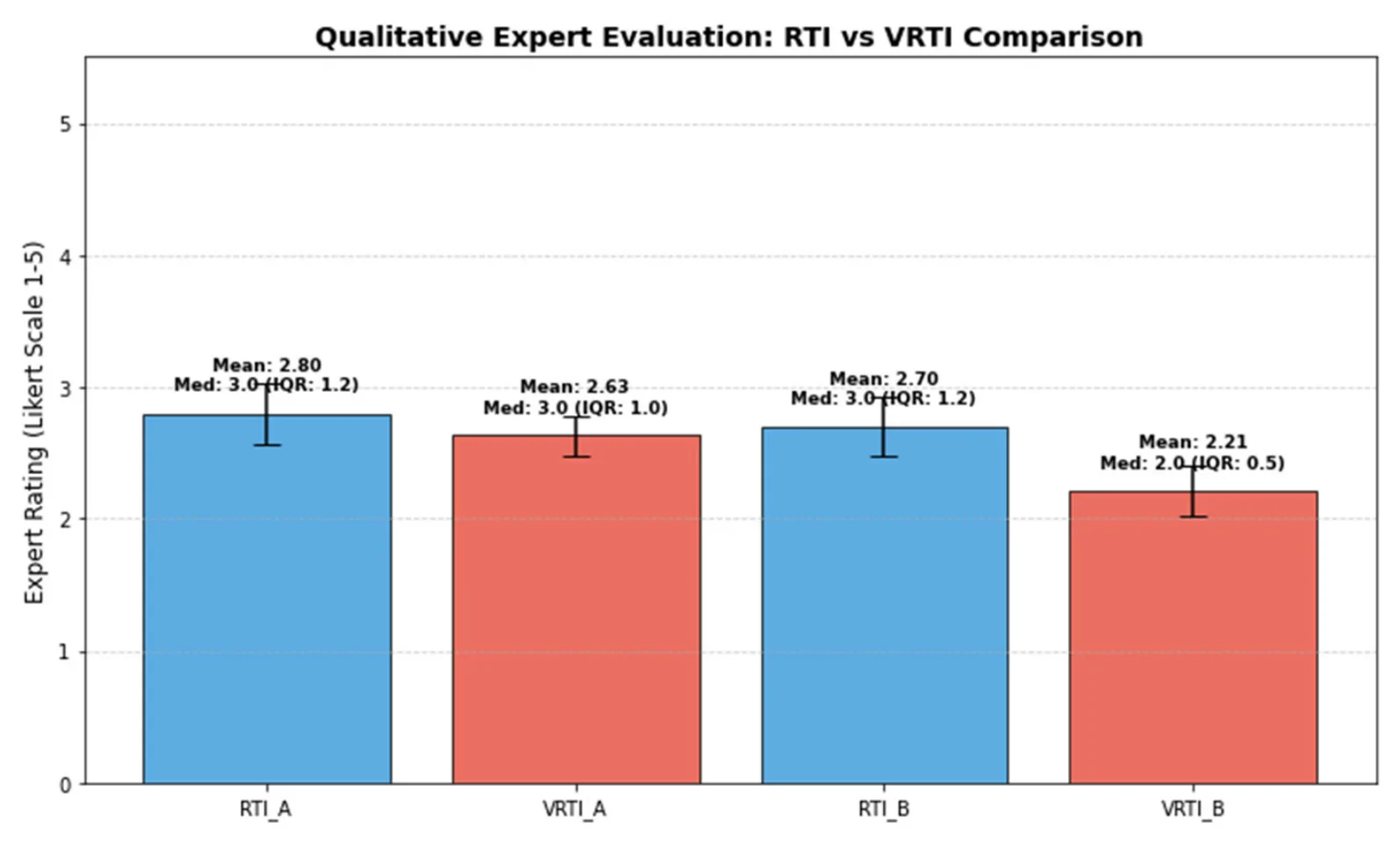
Qualitative expert evaluation results for RTI and VRTI across both tasks.

The qualitative assessment, based on a one-to-five Likert scale provided by a domain expert, corroborates the quantitative trends and is summarized in Figure 15. Outcomes from the test displayed variability across both RTI and VRTI groups and tasks. According to the expert feedback, this result was expected, as sample interpretation was not straightforward, and student participants were interacting with this specific specimen and tasks for the first time.

Given the ordinal nature of Likert-type ratings, the expert evaluations are summarized using non-parametric distribution metrics (median and interquartile range (IQR)) alongside descriptive parametric measures (mean and standard error of the mean (SEM)) embedded directly within Figure 15. No inferential statistical hypothesis testing was performed on these qualitative ratings; rather, differences between RTI and VRTI conditions are interpreted strictly from a descriptive perspective.

For Task A (Footprint), the qualitative ratings indicate a substantial stability in perceived interpretative quality between the two platforms, yielding an identical median score of 3.0 (RTI_A: median = 3.0, IQR = 1.2, and mean = 2.80; VRTI_A: median = 3.0, IQR = 1.0, and mean = 2.63). The short error bars and consistent medians indicate that the expert perceived a comparable level of morphological clarity across both conditions. However, a more pronounced descriptive divergence was observed in Task B (Detail). Tracings produced under the traditional RTI condition received higher qualitative ratings (median = 3.0, IQR = 1.2, and mean = 2.70) than those completed in VRTI (median = 2.0, IQR = 0.5, and mean = 2.21). This discrepancy suggests that the qualitative and quantitative measures may capture different aspects of tracing performance. While the quantitative unidirectional RMSE indicated spatial agreement with the ground truth in VRTI, the expert assigned a lower qualitative score compared with RTI. This assessment may reflect qualitative characteristics of the tracing that are not directly captured by this metric. Complementary bidirectional metrics did not reveal statistically significant differences between the two groups, suggesting that the lower qualitative score assigned to VRTI should not be interpreted straightforwardly as evidence of lower spatial accuracy. The apparent divergence between quantitative accuracy and expert assessment, therefore, represents an aspect requiring further investigation, analyzing whether the observed difference in qualitative scores is specifically related to a more fragmented or cautious tracing style.

### 4.3. Lighting Perspective and Spatial Behavior

**Figure 16:**
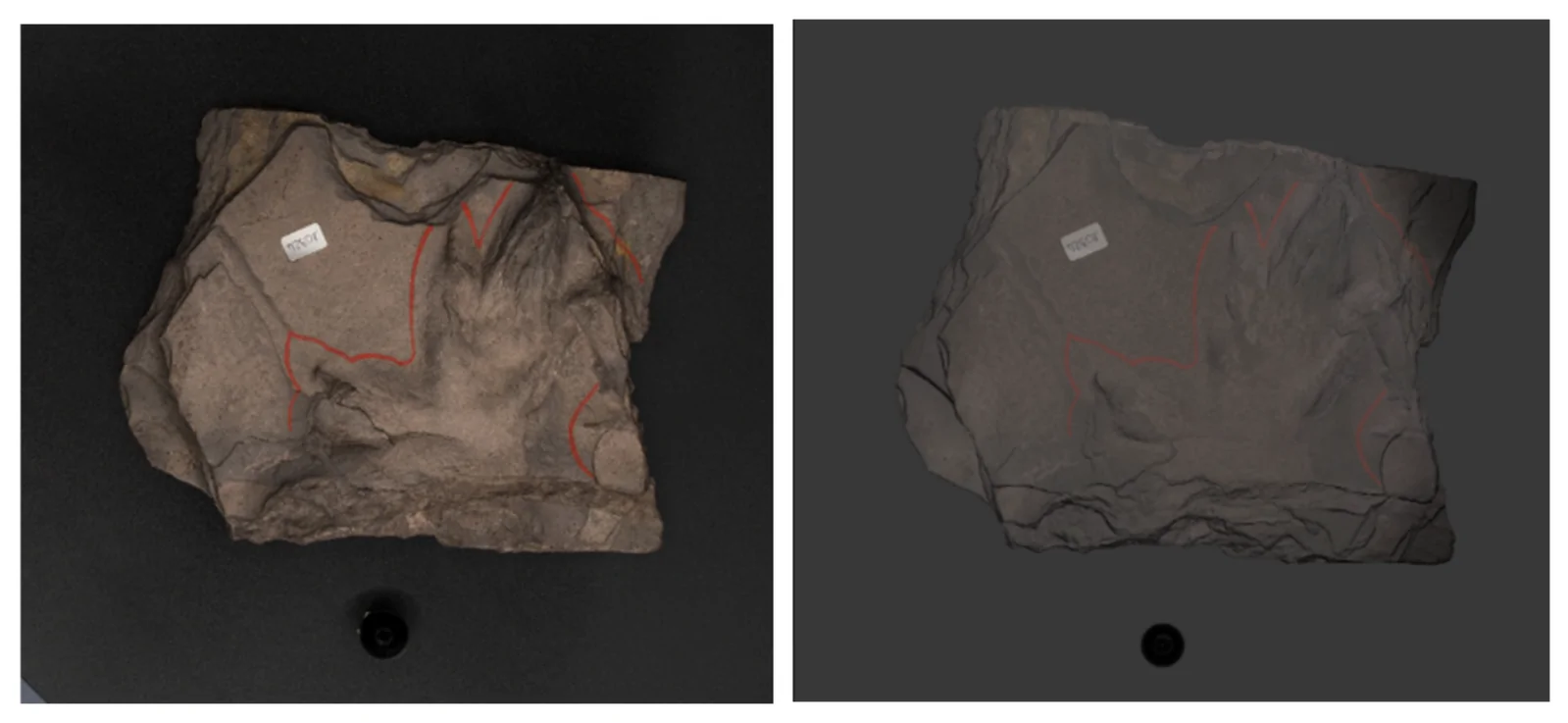
Mean of the chosen best lighting perspective for RTI (**left**) and VRTI (**right**).

The statistical comparison of the selected lighting coordinates is summarized in Table 4. Figure 16 shows the representative lighting perspective generated by applying the mean lighting coordinates (x, y) to the dynamic light source.

**Figure 17:**
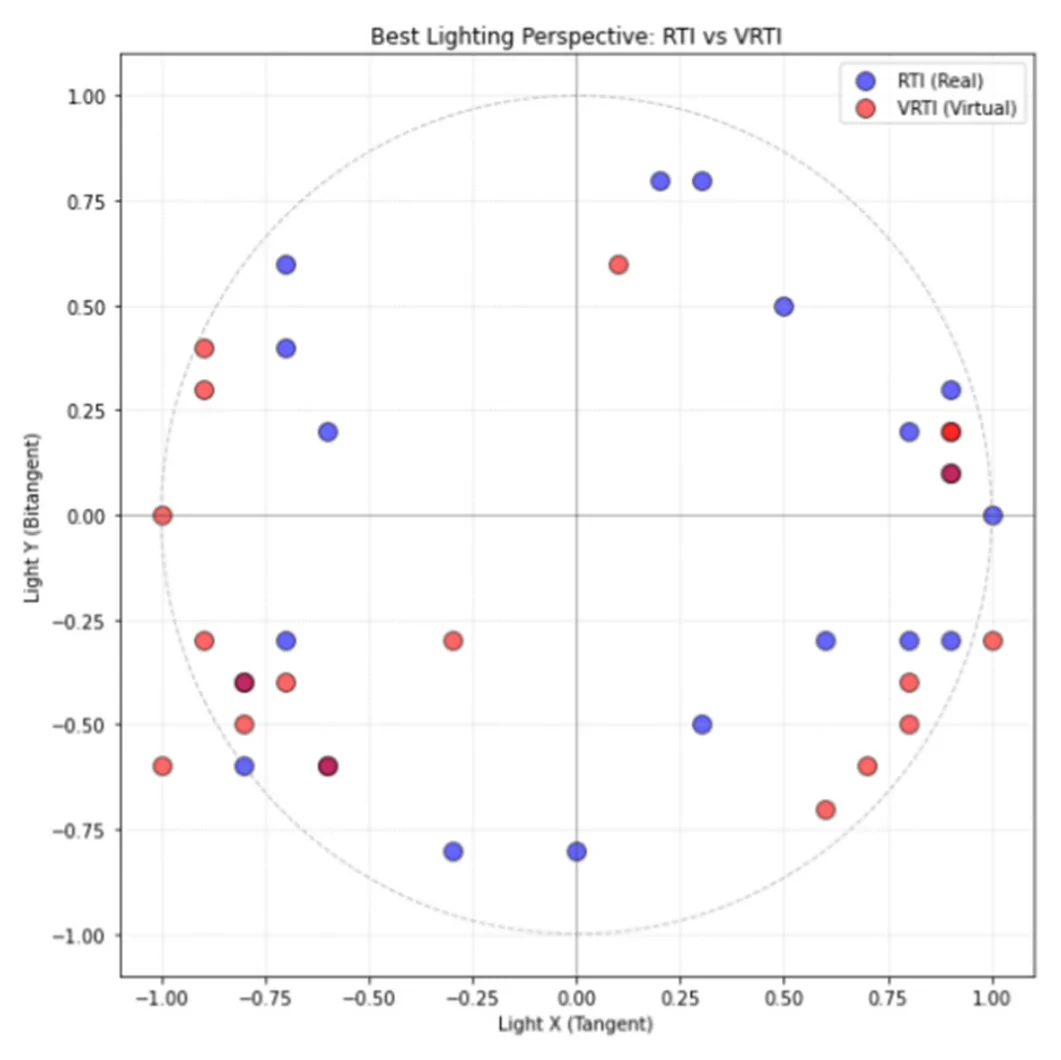
Distribution of chosen dynamic light perspective for morphological identification.

The scatter plot (Figure 17) reveals a high degree of dispersion across the hemispherical domain. The absence of a single, dense “cluster” suggests that there is no universal standard shared point for illumination. Instead, students identified relevant details from a wide variety of angles, indicating that the perception of the artifact’s micro-topography is highly individual. A clear trend is observable as most selections (both RTI and VRTI) gravitate toward the edges of the unit circle (values approaching 1.0 or −1.0). This confirms a natural preference for low-angle grazing light, which maximizes micro-shadows and enhances the legibility of the footprint’s surface relief. The *p*-values (0.36 and 0.34) are significantly above the threshold. This is a key finding: the visualization medium, whether real (RTI) or virtual (VRTI), does not statistically influence the lighting choice, and the overall “spatial behavior” remains comparable across both groups. Given the high variance, the scatter plot proves to be the most effective diagnostic tool.

Finally, to evaluate the potential impact of interactive illumination choices on drawing outcomes, we examined the statistical relationship between the users’ physical interaction with the light dome and their geometric recording errors (RMSE). The Spearman rank correlation analysis revealed no statistically significant relationship across all tested conditions (p≥0.05), as shown in Table 5. These quantitative results indicate that drawing accuracy remained statistically independent of individual lighting angles selected by participants.

### 4.4. SUS Results of VRTI Workspace

**Figure 18:**
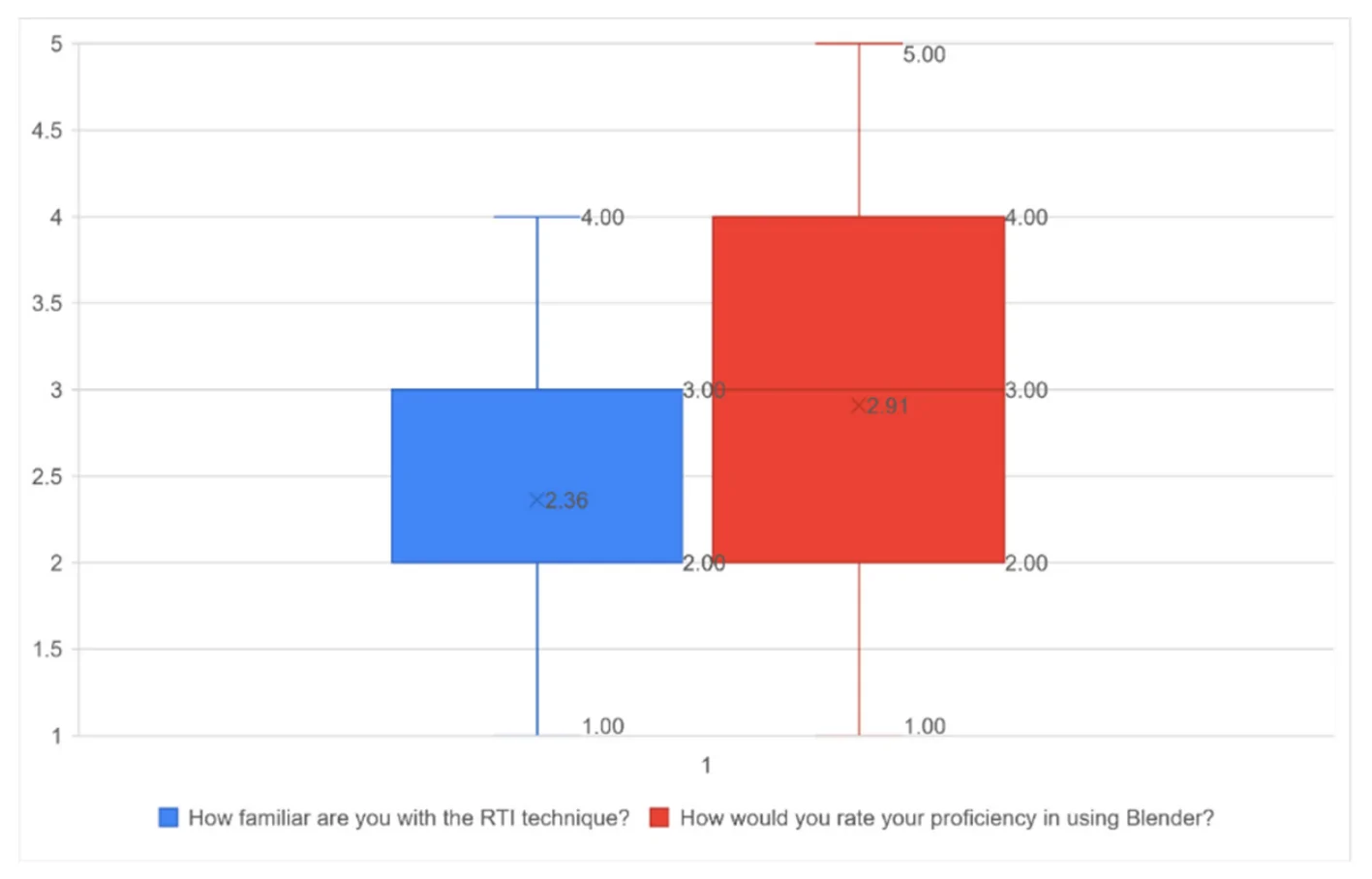
Participants’ familiarity with RTI methodology and Blender environment.

Referring to paragraph 3.6, a pilot usability evaluation of the VRTI workspace in Blender was administered anonymously to a heterogeneous group in terms of proficiency with 3D tools for cultural heritage and RTI applications. The questionnaire begins with two preliminary questions aimed at assessing participants’ prior familiarity with both the RTI methodology and the Blender environment, with ratings ranging from 1 to 5 (Figure 18).

The users involved answered an average of 2.4 to the first question related to RTI and 2.9 for Blender. Given the limited sample size, the results should be interpreted as preliminary evidence aimed at identifying usability trends and potential areas for improvement, with the average being heavily influenced by individual responses, resulting in a high dispersion of scores. In addition to the mean, the median was also calculated: 2 for RTI familiarity and 3 for Blender proficiency, providing a more robust measure of the central tendency. These questions were included to contextualize the usability results and evaluate the potential influence of previous technical expertise on user performance and confidence.

**Figure 19:**
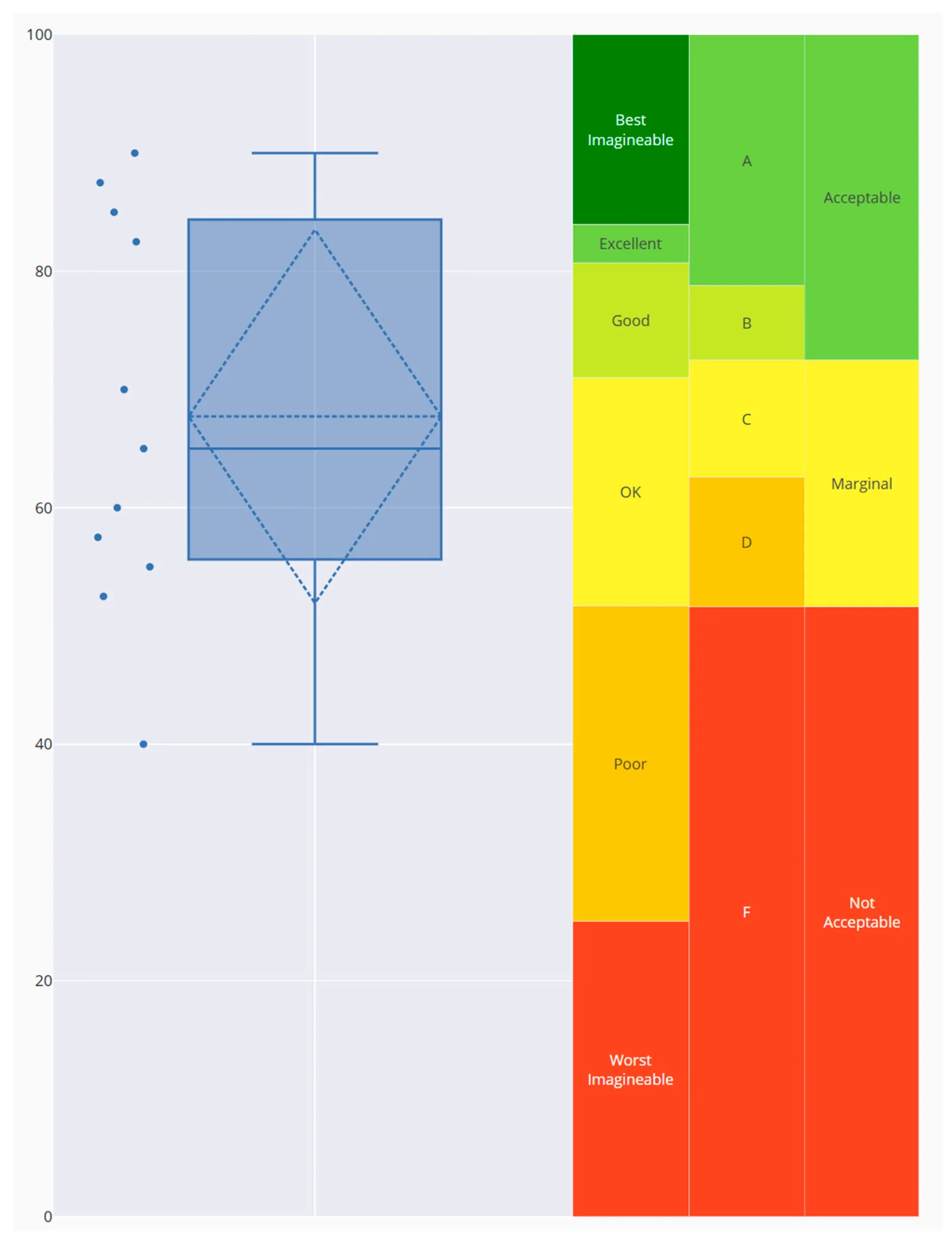
Distribution of SUS scores for VRTI workspace.

Usability was evaluated through the standard 10-item System Usability Scale (SUS) (https://www.surveylab.com/blog/system-usability-scale-sus/, accessed on 26 May 2026). Responses were exported as CSV data and processed using the SUS scoring tool (https://sus.mixality.de/). Following the standard SUS methodology, odd-numbered items were scored using the formula (x − 1), while even-numbered items were scored using (5 − x) due to their negative wording. The resulting values were normalized on a 0–10 scale and converted into a final SUS score on a 0–100 scale. The system obtained an average SUS score of 67.7/100, with a median value of approximately 66–67. According to standard SUS interpretation guidelines, this result indicates an acceptable and slightly above-average level of usability. Nevertheless, the distribution of responses revealed considerable variability among participants: half of the participants scored between 57 and 84; the minimum was 40; and the maximum was 90. The user experience presented a high dispersion in the resulting scores (Figure 19). 

As shown in Table 6, the lowest-scoring items were associated with user confidence and perceived complexity, particularly Questions 8, 9, and 10, as shown in Figure 19. Question 9 (“I felt very confident using the system”) received the lowest normalized contribution score (5.45). Examination of the individual responses suggested that the lowest confidence score was generally associated with lower levels of familiarity with RTI and proficiency in Blender, as shown in Table 7. The lowest confidence scores (2/5) were reported by P5, P9, and P10, who also reported low-to-moderate familiarity with RTI (1–2/5) and Blender (2–3/5). Conversely, participants reporting higher Blender proficiency (4–5/5; P2, P3, and P6) consistently reported high confidence (4/5). However, this pattern was not uniform across the sample, as P1 reported high confidence (4/5) despite minimal Blender proficiency (1/5). 

At the end of the questionnaire, participants were invited to provide optional open-ended comments. The feedback highlighted both the perceived usefulness of the system and the importance of prior Blender expertise. While experienced users described the workflow as clear and straightforward, novice users suggested the inclusion of additional onboarding materials, such as short video tutorials or guided setup instructions, to facilitate the initial learning process. As the open-ended question was optional, qualitative feedback was not provided by all participants and varied in detail. The comments were, therefore, considered supplementary qualitative observations rather than a systematic qualitative dataset (Table 7). Overall, the feedback highlighted specific aspects not directly captured by the SUS, particularly the computational requirements of the rendering process. Other observations concerned Blender-specific operations, such as light scaling and camera configuration, as well as the potential usefulness of additional guidance for less experienced users Table 8. 

Based on these findings, the finalized workspace was made publicly available through a dedicated GitHub repository (https://github.com/frafrabbiu/VRTI_dome, accessed on 1 July 2026) as a reusable FAIR-compliant digital resource. Its publication is intended to facilitate the adoption of the validated VRTI workflow, promote reproducibility, and support its reuse in future cultural heritage and ichnological applications. The repository includes the complete workspace together with a comprehensive README file providing step-by-step instructions for setting up the virtual dome and performing VRTI acquisitions on any 3D model, thereby facilitating both the implementation of the workflow and its reuse by future users.

## 5. Discussion of the Results

The evaluation of RTI and VRTI was structured around two complementary dimensions: an operational dimension, concerning acquisition procedures, workflow efficiency, and practical applicability, and an interpretative dimension, concerning the extent to which each visualization approach supports footprint readability and scientific analysis. 

This distinction allows both the methodological implications of the two techniques and their impact on the interpretative process to be considered. With respect to the operational dimension of RTI and VRTI workflows, the RTI acquisition of the footprints revealed significant acquisition constraints. In the context of the acquisition of the three footprints, the initial dataset suffered from subtle micromovements during data capture, which compromised data consistency and necessitated a complete re-acquisition. This issue, while not unexpected, is particularly relevant given that the acquisition took place in a museum environment, which is considerably more controlled and adjustable than typical excavation contexts where footprints usually are documented. The second acquisition produced a higher number of images (120) and improved texture resolution; however, this outcome was achieved only through an iterative process because, without a specialized setup featuring a structured dome, the result is more prone to human accuracy issues.

In contrast, the VRTI setup shows substantially greater operational simplicity, requiring fewer adjustments and enabling a more stable and user-friendly acquisition workflow for the operator. Moreover, one of its main advantages compared with traditional RTI is that it can be applied retrospectively to existing 3D datasets, including models originally acquired for documentation or research purposes during fieldwork, excavation activities, or subsequent study phases. Considering the supported hypothesis in which the 3D model preserves a sufficiently detailed representation of the surface geometry, VRTI analyses can be replicated without the need for additional acquisition campaigns, thereby extending the scientific and analytical value of already available digital resources.

Regarding the visual dimension, comparative studies are generally focused on technical aspects of image quality, such as rendering fidelity, or the analysis of normal maps derived from the acquisition process. While these approaches provide valuable information on the graphical and geometric characteristics of the resulting visualizations, they offer limited insight into how the visual medium affects the scientific interpretation of paleontological footprints, since such metrics and approaches are not considered diagnostically relevant within ichnological studies. Instead, the comparative analysis between RTI and VRTI was based on footprint readability, thereby addressing the subjective and perceptual visualization dimensions of footprint analysis that are central to ichnological interpretation. Although the results are based on a small number of participants, they suggest a clear consistency in user performance across both techniques. This finding indicates that, for the perceptual tasks evaluated in this study, the visualization medium is transparent to the operator, with interpretative results being minimally influenced by the choice of rendering approach. The combination of spatial data and expert feedback supports the conclusion that VRTI is a perceptually reliable tool and leads us to suspect that, in the field of footprint research, the human interpretative factor may have a significantly greater influence than the technological tool itself. In this context, while using a single domain expert provided deep, specimen-specific contextual insights, we acknowledge that this setup introduces potential evaluator bias and limits inter-rater reliability analysis. Consequently, this qualitative layer is framed as a preliminary, expert-guided assessment designed to contextualize the quantitative findings rather than a generalized perceptual trial. As emphasized throughout this study, a degree of subjectivity is inherent to paleontological interpretation—whether conducted by a senior specialist or a trainee—because morphological readability depends heavily on individual perception and feature prioritization under varying lighting conditions. Consequently, the observed divergence between quantitative accuracy metrics and expert scores may partly reflect individual interpretative nuances rather than fundamental structural differences between RTI and VRTI. 

In Task B, therefore, the error rates that vary widely (from 20 to over 100 pixels) within the same experimental group may be associated with the complexity of the morphological details, posing a cognitive challenge that transcends the digital interface. The wide range of performance highlights that when a subject is difficult to interpret (e.g., the fine details of a digital pad), the individual’s subjective perception and experience become the dominant variables. The absence of statistically significant differences (Mann–Whitney U; *p* > 0.05) suggests that the VRTI workflow is perceptually transparent for the tasks evaluated in this study, indicating that the technology integrates seamlessly into the user experience without measurably affecting task performance. Although the VRTI data are a synthetic reconstruction, no evidence of perceptual bias or geometric distortion emerged from the analyses performed. Participants appeared to adopt comparable interpretative behaviors when analyzing both RTI and VRTI, suggesting that the high-resolution 3D surface preserves the critical information needed for paleontological documentation.

This human-centric finding seems to be further supported by the lighting perspective analysis. The extreme subjectivity in choosing the “best” light angle (with choices scattered across the entire hemispherical domain) reinforces the idea that there is no universal technological solution for perfect visibility. The ability to identify paleontological features remains a dialogue between the observer’s eye and the light, regardless of whether that light is calculated from physical reflectance or simulated through a virtual environment. Furthermore, the potential correlation between individual lighting choices (whether RTI or VRTI) and tracking performance does not emerge as a significant factor; on the contrary, the results would seem to suggest that the accuracy of interpretation does not depend on specific lighting strategies.

The quantitative findings support the interpretation that the technology is transparent with respect to interpretative accuracy. Meanwhile, the lower qualitative scores from the expert and the omissions seen in the bidirectional data suggest that the virtual medium may reflect a more cautious or fragmented interpretative behavior. This may be due to the subtle differences in how shadows are rendered on a 3D mesh compared with a traditional RTI file, leading students to only trace what they felt was absolutely certain. The observed divergence may result not only from the different visualization paradigms, but also from differences in the underlying data acquisition and representation. RTI was generated from photographic captures under controlled lighting conditions, whereas VRTI combines a 3D surface acquired through structured-light scanning with texture information derived from the scanning process. Therefore, the present experimental design does not allow the effects of the visualization method to be fully disentangled from those of the underlying geometry and texture representation. A deeper analysis would, therefore, require the same evaluation protocol to be applied to a hybrid VRTI workflow combining structured-light scanning with photogrammetry, allowing the contribution of texture photorealism to footprint readability and visual interpretation to be assessed separately.

However, while the expert perceived a more ‘hesitant’ style in VRTI at a descriptive level, the quantitative data provide a complementary perspective: the bidirectional metrics show comparable levels of omission-related error across the two platforms (RTI: 187.52; VRTI: 188.45). This suggests that part of the observed variability may lie in the cognitive and perceptual interpretation of the fossil itself (the “eye of the observer”) rather than being systematically determined by the visualization medium. More broadly, this finding may reinforce the importance of considering both geometric accuracy and human interpretation when evaluating visualization methods for paleontological analysis. The apparent divergence between the two types of assessment does not necessarily indicate a contradiction but rather highlights the complementary information provided by quantitative spatial metrics and descriptive expert-based evaluation. In this respect, the VRTI results appear to preserve the relevant information required for footprint readability and visual interpretation, without evidence of a systematic additional effect on tracing performance. 

Despite the consistency of these results, the limited sample size of n ≈ 20 participants per group makes this study sensitive to individual variability. Although the Mann–Whitney U test did not identify statistically significant differences between the groups, larger and more diverse samples would provide greater statistical power and help determine whether the trends observed in Task B warrant further investigation.

The results of the comparative evaluation provided the methodological basis for the subsequent usability assessment of the Blender workspace, providing encouraging evidence for the use of VRTI as a complementary visualization approach. The SUS evaluation was, therefore, conducted to assess whether the proposed workflow could also meet the practical requirements of scalability, accessibility, and adoption by cultural heritage professionals. In terms of the usability of the workspace for VRTI, the SUS evaluation suggests, at a pilot study level, that the proposed system achieves a satisfactory level of usability and integration within the Blender environment, while also highlighting the need for additional guidance for users with limited experience in 3D software workflows. The results indicate a generally positive perception of the interface, although a certain degree of variability emerged among participants’ evaluations. The aspects most positively perceived by participants were the usefulness of the workspace and its learnability after an initial familiarization period. The main usability issues were reported by users with lower proficiency in Blender, who tended to report lower confidence, while the evaluation also highlighted potential difficulties in navigating and managing Blender-specific functionalities, which may increase occasional disorientation. Previous technical experience may, therefore, represent a factor associated with perceived confidence, although the small sample size does not allow this relationship to be generalized beyond this preliminary descriptive observation.

Overall, the workspace can be considered functional and sufficiently usable; the findings also suggest that further refinements could improve accessibility, reduce cognitive complexity, and support a higher overall level of usability. However, the SUS assessment primarily captures perceived usability and does not provide a direct quantitative measure of operational efficiency, such as processing time, operator effort, or the number of workflow steps required to complete a task. These aspects, therefore, remain to be quantitatively assessed in future comparative studies. However, the sample size is very limited (*n* = 11), which restricts the reliability and generalizability of the findings. The results should, therefore, be interpreted as preliminary evidence from a pilot usability assessment rather than as a robust validation of the operational efficiency of the proposed workflow. For robust and statistically sound conclusions, the sample would need to be substantially expanded. 

### Future Works

To overcome the statistical limitations associated with small sample sizes, future research should first focus on expanding both evaluation cohorts to minimize individual variability. The preliminary effect sizes established in this study ($|r_{rb}|\approx 0.20$) provide an empirical baseline for future a priori power analysis, helping to determine the precise sample sizes required to detect prespecified effect sizes with adequate statistical power, particularly for tasks involving fine morphological interpretation. Furthermore, the preliminary usability assessment (*n* = 11), currently framed as a pilot study, will be scaled up to a larger and more balanced participant pool to improve the generalizability of the usability findings.

The qualitative evaluation should also be expanded beyond the single domain expert involved in the present study. While expert assessment was included to contextualize the quantitative metrics from a paleontological perspective, relying on a single evaluator introduces inherent subjectivity. Since paleontological interpretation naturally involves individual visual perception, future research will expand this qualitative framework to include a broader panel of independent evaluators, ranging from students to senior experts. This will enable inter-rater reliability analyses to quantitatively assess evaluator agreement and further decouple subjective interpretative bias from objective dataset quality. 

More specifically, future studies should investigate the sources of individual variability observed in the more demanding detail-tracing task. Dedicated error-pattern analyses, including tracing continuity, omission patterns, and the spatial distribution of errors, could help clarify whether differences in performance are associated with specific morphological features or tracing strategies. These analyses could be complemented by participant-level covariates, such as prior experience with 3D visualization or digital modelling, as well as by a more systematic characterization of morphological complexity. In parallel, individual interpretative variability should be considered alongside technical factors that may influence VRTI perception. Comparative VRTI workflows using different texture acquisition strategies, including combinations of structured-light scanning and photogrammetry, could help disentangle the contribution of texture characteristics from that of the 3D visualization environment. Controlled experiments with larger samples varying lighting, rendering conditions, and rendering engines would further clarify whether specific properties of the virtual representation influence perceptual and interpretative performance. 

The comparative protocol should also be replicated across a broader range of fossil track specimens, including different ichnogenera, preservation conditions, morphological complexity, and host-rock lithologies. Such cross-specimen validation would help assess the robustness and generalizability of the observed RTI–VRTI performance across different ichnological contexts.

Finally, the operational advantages attributed to VRTI require dedicated quantitative assessment. The present study did not directly measure processing time, operator effort, or workflow efficiency. Future comparative studies should, therefore, assess processing and task completion times under controlled hardware and software conditions, while also considering measures such as the number of user interactions or workflow steps, error rates, and perceived workload. These measures would provide a more objective basis for evaluating the operational efficiency and usability of VRTI in comparison with conventional RTI workflows. 

## 6. Conclusions

This study contributes to the ongoing development of standardized and reproducible workflows for the analysis of paleontological footprints by introducing both a FAIR-compliant VRTI workspace and a structured protocol for comparing RTI and VRTI visualizations. RTI has proven to be a valuable tool for documentation of paleontological footprints. However, despite its widespread adoption, the lack of a standardized methodological framework for analysis and comparative interpretation remains a significant limitation. This study introduced structured evaluation protocols aimed at addressing key open challenges and defining a reproducible workflow for VRTI within a FAIR-compliant, open-source environment implemented in Blender. The comparative evaluation protocol [27] between RTI and VRTI showed comparable performance in terms of footprint readability, particularly under raking-light conditions, supporting its central role in ichnological visualization workflows. Importantly, statistical analysis revealed no significant differences between the two modalities (Mann–Whitney U; *p* > 0.05), with the findings being consistent with comparable perceptual performance and providing no evidence of a systematic reduction in interpretative accuracy in the VRTI condition. Overall, the results suggest that individual variability may play a crucial role in the interpretation of complex morphological features, although the contribution of technical and acquisition-related factors cannot be excluded.

The proposed VRTI workspace showed improved configurability and scalability across different acquisition scenarios, supported by standardized naming conventions and metadata structures to ensure reproducibility. The usability assessment (SUS) indicates an overall acceptable level of usability, although results from the pilot study (*n* = 11) highlight variability linked to prior experience with 3D software and suggest the presence of disorientation during initial interaction, as well as the need for further testing with a larger and more diverse user base. These challenges could be mitigated through the integration of additional tutorials and supporting documentation. Ultimately, while the interpretation of footprints and tracks in ichnology inherently retains a degree of subjectivity, VRTI offers a promising complementary analytical tool for revising and comparing published material when 3D models are available. An important strength of VRTI lies in its ability to be applied retrospectively to existing high-resolution 3D datasets, enabling new analyses without requiring additional acquisition campaigns and, thus, maximizing the long-term scientific value of digital archives. This capability is particularly valuable when field acquisition conditions are less than optimal, as virtual relighting can enhance feature visibility and support more effective interpretation.

Although the results provide encouraging evidence for both the scientific validity of VRTI and the usability of the proposed workspace, further validation involving larger and more diverse participant groups, together with a broader range of ichnological datasets, will help consolidate the statistical robustness and generalizability of the proposed methodology. Future studies should also investigate the contribution of individual participant characteristics, morphological complexity, texture acquisition quality, lighting conditions, and rendering parameters to VRTI interpretation. Comparative experiments using alternative acquisition and texturing strategies, including hybrid workflows combining structured-light scanning and photogrammetry, together with dedicated analyses of tracing continuity, omission patterns, and error distributions, could further clarify the factors underlying differences between RTI and VRTI performance. This line of research will contribute to the consolidation of a standardized VRTI workflow and support its potential extension beyond paleontological applications into the broader field of cultural heritage documentation and analysis. 

## Figures and Tables

**Table 1:** Summary of 3D digitization and virtual rendering approaches in VRTI studies.

Study	Digitization	Virtual Dome Rendering
[2]	SfM photogrammetry; processing in Agisoft PhotoScan	Cinema 4D; virtual lighting + virtual reflective target; rendering of image sequence
[4]	Structured-light scanning (Artec Eva; Artec Studio Professional)/close-range photogrammetry (Agisoft PhotoScan Professional)	Cinema 4D; 54-light virtual hemispherical rig; automated rendering via WinParrot
[17]	High-resolution 3D surveys from photogrammetry and scanning	Blender/Cycles; 54-light virtual dome; Python script for sequential automated rendering
[3]	SfM photogrammetry; image preprocessing in Adobe Camera Raw; 3D reconstruction in Agisoft Metashape	Blender; 49-light virtual dome; reflective sphere; automated rendering through Blender animation
[16]	Underwater photogrammetry of archeological blocks	3ds Max 2024; 101-light virtual dome + reflective sphere; two scripts control lighting and rendering
[5]	TLS, Trimble TX6; initial conversion in Trimble RealWorks; CloudCompare + MeshLab for processing/meshing	Blender; normal-map baking + 49-light virtual dome; EEVEE; automated rendering

**Table 2:** Acquisition and 3D model metadata of the three footprints (CNA1943, CNA1944, and CNA1946).

Scanner: Artec Spider II	Source Scans	Maximum Error (Resolution)	Tracking Mode	FPS	Triangular Mesh	3D Model Size
CS1 (CNA1943)	10	0.2 mm	Geometry + texture	8	2,900,000	65 MB
CS2 (CNA1944)	8	0.2 mm	Geometry + texture	8	2,570,807	56 MB
CS3 (CNA1946)	3	0.2 mm	Geometry + texture	8	2,997,132	65 MB

**Table 3:** Summary of quantitative metrics (RMSE, P90, and StdDev) for footprint (Task A) and detail (Task B) tracing. All values are expressed in pixels. The *p*-values refer to the Mann–Whitney U test comparison between RTI and VRTI conditions.

Task	Group	RMSE	P90	StdDev	*p*-Value
A (Footprint)	RTI	57.30	96.86	42.42	0.361
	VRTI	59.70	104.98	43.36	
B (Detail)	RTI	67.04	123.85	47.41	0.255
	VRTI	59.46	105.54	37.96	

**Table 4:** RTI and VRTI mean and median metrics in best lighting perspective selection.

Axis	RTI Mean	VRTI Mean	RTI Median	VRTI Median	*p*-Value
X	0.10	−0.06	0.25	−0.30	0.366
Y	−0.05	−0.20	−0.15	−0.30	0.344

**Table 5:** Correlation metrics between lighting angles and tracing accuracy (RMSE).

Condition	Task	Spearman r_s_	*p*-Value
RTI	A	−0.171	0.471
	B	−0.121	0.610
VRTI	A	0.119	0.628
	B	−0.205	0.400

**Table 6:** SUS item responses (Likert scale of 1–5) and normalized average results (0–10).

SUS Question	Normalized Average Values (0–10)
1. I think that I would like to use this system frequently	6.36
2. I found the system unnecessarily complex	7.73
3. I thought the system was easy to use	6.82
4. I think that I would need the support of a technical person to be able to use this system	7.27
5. I found the various functions in this system were well integrated	7.73
6. I thought there was too much inconsistency in this system	7.73
7. I would imagine that most people would learn to use this system very quickly	6.36
8. I found the system very cumbersome to use	6.14
9. I felt very confident using the system	5.45
10. I needed to learn a lot of things before I could get going with this system	6.14

**Table 7:** Individual participants’ prior familiarity with RTI and Blender and perceived confidence in using the VRTI workspace. Confidence corresponds to the original Likert-scale response (1 = strongly disagree; 5 = strongly agree) to SUS Question 9 (“I felt very confident using the system”).

Participant	How Familiar Are You with the RTI Technique?	How Would You Rate Your Proficiency in Using Blender?	9. I Felt Very Confident Using the System
P1	3	1	4
P2	3	5	4
P3	2	4	4
P4	4	3	3
P5	2	3	2
P6	3	4	4
P7	2	3	4
P8	3	3	3
P9	1	2	2
P10	1	2	2
P11	2	2	3

**Table 8:** Qualitative feedback on the VRTI workspace from the optional open-ended question.

Participant	Additional or More Detailed Feedback, Including Suggestions for Improvement, Would Be Greatly Appreciated.
P1	I only did a quick test and imported the shots into Relight, and it feels really effective (if I had found this workspace in a new version of Blender, I would’ve thought: “wow cool”). The instructions are clear and easy to follow. Maybe you could add a small note at the bottom that “permanently” shows when the render is completed.
P2	It took me a couple of hours to get the renders, but that certainly depends on my computer. Otherwise, it seems to work really well.
P3	The system is very easy to use. The instructions are clear and the workflow is simple to follow. Overall, it is a very useful tool for research. Congratulations.
P4	I think the system is a good one and my responses are more a reflection on my own knowledge/experience with Blender combined with the READ ME instructions. This system wouldn’t be a problem at all for expert Blender users but for novice ones (like me) I wasn’t very familiar with adjusting the lights scale and camera settings. I wasn’t even sure if I should adjust the lights’ scale based on the size of my object. Some short video tutorials for the setup (and why it matters) would improve usability a lot for these blender-specific tasks. But the script itself worked fine, and it was easy to execute based on the Readme.
P5	In order to use the tool, it is necessary to have an entry-to-mid knowledge of Blender and a significantly powerful computer

## Data Availability

The PTM files used in this study are publicly available at ref. [34]. The raw RTI and VRTI files, as well as the experimental datasets generated and analyzed during the current study, are not publicly available because this work reports the results of a pilot study with a limited number of participants. The data are available from the corresponding author upon reasonable request.
